# Chronic respiratory diseases risk during the COVID-19 pandemic: an integrated modelling approach based on hospital records across 30 countries

**DOI:** 10.1186/s12963-025-00412-x

**Published:** 2025-11-06

**Authors:** Cui Zhou, Jing Gao, Pia Lindberg, Chutian Zhang, Yuchen Wang, Jian Ma, Åsa M. Wheelock, Lei Xu

**Affiliations:** 1 Vanke School of Public Health, Tsinghua University, Beijing, 100084 China; 2 Institute for Healthy China, Tsinghua University, Beijing, China; 3 Division of Immunology and Respiratory Medicine, Department of Medicine Solna, Karolinska Institutet, Stockholm, Sweden; 4 Department of Respiratory Medicine and Allergy, and Center for Molecular Medicine, Karolinska University Hospital, Stockholm, Sweden; 5 Department of Respiratory Medicine, Gansu Provincial Hospital, Lanzhou, China; 6 College of Natural Resources and Environment, Northwest A&F University, Yangling, 712100 China; 7 Institute of Plant Protection, Henan Academy of Agricultural Sciences, Zhengzhou, 450002, China

**Keywords:** Prehospital risk, Chronic respiratory disease, Asthma, Risk stratification, COVID-19

## Abstract

**Background:**

Globally, there are significant inequalities in risk for chronic respiratory disease patients with COVID-19 (CRD-COVID), and a comprehensive understanding of its determinants and their interactions is needed. This study quantified individual, environmental, and viral risks that impact hospital admission severity and survival outcomes in CRD-COVID patients utilizing multinational hospital records.

**Methods:**

We analysed data on CRD-COVID from the International Severe Acute Respiratory and emerging Infection Consortium (ISARIC) dataset, covering January 2020 to July 2022 across 30 countries. The cohort included COVID-19 patients with asthma (Asthma, n = 36,365), chronic pulmonary disease (CPD, n = 36,332), and asthma-CPD overlap (ACO, n = 16,061). We matched these patients with their prehospital environmental and viral risk factors. The primary outcome was admission severity, which we assessed using generalised linear mixed models (GLMM), and GPBoost with Shapley Additive Explanations (SHAP) algorithm. The secondary outcome was 28-day mortality, evaluated using Cox regression and K-medoids clustering.

**Results:**

The rates of severe admissions and 28-day mortality were 33.7% and 16.4% for the asthma cohort, 30.1% and 31.6% for the CPD cohort, and 15.9% and 25.8% for the ACO cohort, respectively. Common key risk factors impacting admission severity in CRD-COVID patients include age, sex, comorbidities, humidity, precipitation, and O_3_ concentration, while vaccination status, temperature, and SO_2_ concentration were only significant in asthma patients. The interactions analysis showed low Humidity had a greater impact on patients over 60 years of age and those with comorbid hypertension. Individual, environmental, and viral factors accurately predicted admission severity, and their contribution was different for asthma (58% individual, 28% environmental, and 14% viral variants), CPD (57%, 33%, and 10%) and ACO (63%, 31%, and 6%) patients. Four clusters stratified by these risk factors within each disease group showed significant differences in 28-day mortality rates, particularly in the asthma and CPD patients. The cluster with the highest 28-day mortality rates featured low humidity (mean 55.5% for asthma, 54.4% for CPD) and older age (60.1 and 74.2 years).

**Conclusion:**

The impact of prehospital individual, environmental, and viral risk on the severity of CRD-COVID patients was heterogeneous. Older people exposed to low humidity were at greatest risk.

**Supplementary Information:**

The online version contains supplementary material available at 10.1186/s12963-025-00412-x.

## Introduction

The COVID-19 pandemic poses new threats to the management of chronic respiratory diseases (CRDs) such as asthma. This is due to the fact that patients with CRDs accounted for a relatively large proportion of COVID-19 admissions (ranging from 2.8% to 28.6%) [1], and were more likely to develop varying degrees of severe outcomes compared to those without CRDs [2–5]. Differences in vulnerability among CRD patients that may be attributed to a combination of risk factors, such as individual health status, environmental conditions, viral transmission characteristics, and public health strategies.

Prehospital risk factors play an important role in predicting CRDs exacerbation and COVID-19 adverse outcomes. In particular, the effects of prehospital environmental factors such as air pollutants and relative humidity is of increasing concern [6]. In addition, age, sex, comorbidities, smoking, and viral infections have been widely recognised as individual factors affecting outcomes in this population [4, 7]. Recent hypotheses propose that short-term exposure to air pollutants may increase the risk of exacerbations of CRDs, ultimately leading to their severe COVID-19 outcomes [6]. Long-term exposure to fine particulate matter (PM_2.5_) has been shown to be associated with increased hospitalisation rates in COVID-19 patients with pre-existing asthma [8]. However, despite these observations, there remains a gap in our understanding of prehospital individual and environmental risk factors, as well as their interactions. Therefore, a comprehensive understanding of these prehospital risk factors and their impact on disease severity and progression, and their role in disease stratified management, is needed to bridge the existing knowledge gaps for early prevention of adverse outcomes in CRD patients in the pandemic.

The spatiotemporal data that surged globally during the COVID-19 pandemic provided strong support for an in-depth exploration of risk factors in CRD patients. The International Severe Acute Respiratory and Emerging Infections Consortium (ISARIC) database provides hospitalised individual data on CRD patients infected with COVID-19 (CRD-COVID) from 30 countries to explore their individual and geographical heterogeneity [9]. Additionally, the improved machine learning algorithm, tree boosting with Gaussian processes and mixed effect models (GPBoost), allows taking into account differences between countries, thereby improving the accuracy and reliability of cross-country risk identification and outcome prediction [10].

In this study, we focused on multidimensional prehospital risk factors and explored how they affect the severity of CRD-COVID patients at admission and 28-day mortality, and consequently how they affect clinical progression and risk stratification. We integrated individual health status, environmental conditions and viral exposure to guide early personalised prevention and management strategies for CRD patients in the context of acute respiratory infectious disease epidemics.

## Materials and methods

More details on the participants, data sources and methods are provided in supplementary methods and supplementary tables.

### Participants

This retrospective cohort study is a secondary analysis of the ISARIC COVID-19 dataset, which prospectively collects clinical data on COVID-19 hospitalisations worldwide. The data collection methods and characteristics have been described elsewhere [9]. The ISARIC-WHO Clinical Characterization Protocol received approval from the WHO Ethics Review Committee (RPC571 and RPC572). This study included all CRD-COVID patients (from 30 countries, n = 88,758) with confirmed or suspected SARS-CoV-2 infection form ISARIC dataset from January 2020 to June 2022. The case definitions for CRD-COVID were in line with those used in previous research based on the ISARIC dataset [4], including 1) hospitalised asthma patients infected with COVID-19 (asthma, n = 36,365), 2) hospitalised chronic pulmonary diseases patients (non-asthma) infected with COVID-19 (CPD, n = 36,332), and 3) hospitalised overlapping asthma and CPD patients infected with COVID-19 (ACO, n = 16,061). The category of “chronic pulmonary diseases (non-asthma)” includes a wide range of conditions, such as Chronic obstructive pulmonary disease and bronchiectasis. Patients with incomplete age or sex data or uncertain residence were excluded from the study.

### Outcomes

The primary outcome of interest in our study was severity at admission. We employed a classification method based on the oxygen support requirement at admission, using it to operationalize “disease severity at admission”. The aim was to evaluate the severity of illness upon hospital entry to predict early resource needs and short-term prognostic factors. This measure does not capture clinical progression after admission. Recorded oxygen treatments included “oxygen by mask or nasal prongs”, “high flow oxygen nasal cannula”, “noninvasive ventilation”, “artificial respiration” and “Extra corporeal membrane oxygenation”, and the dates when these treatments were administered. We categorised patients according to the World Health Organisation (WHO) Clinical Progression Scale [11]. As all patients in this study were hospitalized, the terms ‘Moderate’ and ‘Severe’ are used to describe initial disease severity at admission. The secondary outcome was 28-day mortality, which considered whether death occurred at 28 days of admission and the time to death within 28 days. The reported date of admission was taken as day one and discharge from hospital was considered an absorbing state (once discharged, patients were considered to no longer be at risk of death) [12].

### Variables

Individual variables were obtained from the ISARIC dataset and included age, sex, ethnicity, whether or not a health worker, whether or not smoking, whether or not vaccinated against COVID-19, and whether or not each comorbidity was comorbid. Comorbidities include 37 diseases including hypertension, diabetes, chronic cardiac disease, chronic kidney disease, etc., from the case report form. Charlson Comorbidity Index (CCI) was calculated with an ad hoc modified formula (supplementary methods) [13].

Environmental variables included four meteorological conditions and five air pollutant. We extracted their monthly averages to capture short-term exposure [14], and matched them to each patient by country and admission time. Meteorological factors included temperature, precipitation, relative humidity, and wind speed [15, 16]. Air pollutants included sulfur dioxide (SO_2_), nitrogen dioxide (NO_2_), ozone (O_3_), and particulate matter (PM_10_ and PM_2.5_) [17]. All environmental data are pre-processed with population weighting [18, 19].

Each patient was matched to the variant most likely to be infected, based on the SARS-CoV-2 variant with the highest prevalence percentage in each country for that month, to adjust for the impact of different dominant SARS-CoV-2 variants at different times of the pandemic. The variant strains were considered only for the five variants of concern (VOCs) including Alpha, Beat, Gamma, Delta, Omicron and other [20, 21].

### Statistical analysis

#### Generalized linear mixed effect models (GLMM)

GLMM were used to estimate the effects of risk factors on severity at admission. The effects of each environmental factor were estimated separately, all adjusted for all individual factors. Country was introduced as a random intercept to adjust for unquantifiable between-country differences [22]. A logit link function was used to model binary outcomes. Considering interactions between individual and environmental factors, construct interaction terms between each environmental and individual factor individually, keeping those that are statistically significant and have practical explanations or theoretical plausibility. We performed sensitivity analyses to exclude patients with unknown comorbidities. Adjusted odds ratios with 95% confidence intervals (CIs) were presented from multivariable models. P-value < 0.05 was considered statistically significant. Significant variables were included in subsequent multivariate prediction models.

#### GPBoost models and SHapley Additive exPlanations (SHAP) algorithm

We used GPBoost to predict the severity at admission and 28-day mortality, and used SHAP to quantify the contribution of associated factors. GPBoost is a machine learning model that combines tree-boosting with Gaussian processes and mixed effects models [10]. It aims to leverage the advantages of tree-boosting algorithms including accounting for complex nonlinearities, discontinuities and higher order interactions with the versatility of Gaussian processes [23]. We used the recursive feature elimination (RFE) algorithm to filter the features that have significant effects in the GLMM, with the goal of retaining as few factors as possible while achieving a high level of accuracy (supplementary methods, supplementary table S1) [24]. For hyperparameter tuning, the best combination of hyperparameter values was selected using a five-fold cross-validation grid search (supplementary methods). The accuracy of the model was evaluated in terms of AUC. The detailed modelling process is shown in the supplementary file. Finally, SHAP algorithm was used to quantify the importance of each factor. SHAP is a game theory based machine learning post-hoc explanation algorithms that quantifies the contribution of each factor in the model [25]. Relative importance scores for each factor affecting severe admission and 28-day mortality in the models, obtained by taking the absolute mean of the SHAP values.

#### K-medoids clustering

The stratification of subgroups within each category of CRD-COVID was analysed using the unsupervised clustering k-medoids method [26]. Classification was based on predicted COVID-19 severity at admission, clustering patients in the severe group and patients in the moderate group separately. The values of all key predictor variables were standardized. The ‘manhattan’ distance metric was used and the optimal number of clusters was selected using the silhouette coefficients. Cluster membership was determined for each individual.

#### Survival analysis and other statistical methods

The Kaplan–Meier survival estimates were used to compare 28-day mortality across subgroups. The Cox proportional hazards model after checking proportional hazards assumptions was used to test the association between subgroup type and final outcome as well as length of hospitalization. To describe the characteristics of each cluster, continuous data were expressed as means with standard deviation (SD), and categorical variables were summarised as percentages of categories. Comparisons of continuous and categorical variables between categories were performed using analysis of variance (ANOVA) and chi-square tests with post-hoc tests using the Bonferroni method.

#### Sensitive analysis

To account for potential biases related to clinical protocols and Healthcare resource constraints during the early phase of the pandemic, we conducted a sensitivity analysis by excluding data from the first quarter of 2020. We re-estimated the associations between individual and environmental factors and disease severity at admission after excluding these early cases. Additionally, we re-trained the GPBoost prediction model on the dataset excluding early cases and compared the change in AUC to assess the robustness of the model’s predictive performance.

For statistical analysis, we utilized the ‘gpboost’, ‘cluster’, and ‘stats’ packages in R (version 4.3.0), and ‘sklearn’ and ‘gpboost’ in Python (version 3.10).

## Results

### Characteristics and outcomes of the CRD-COVID cohort

**Fig. 1:**
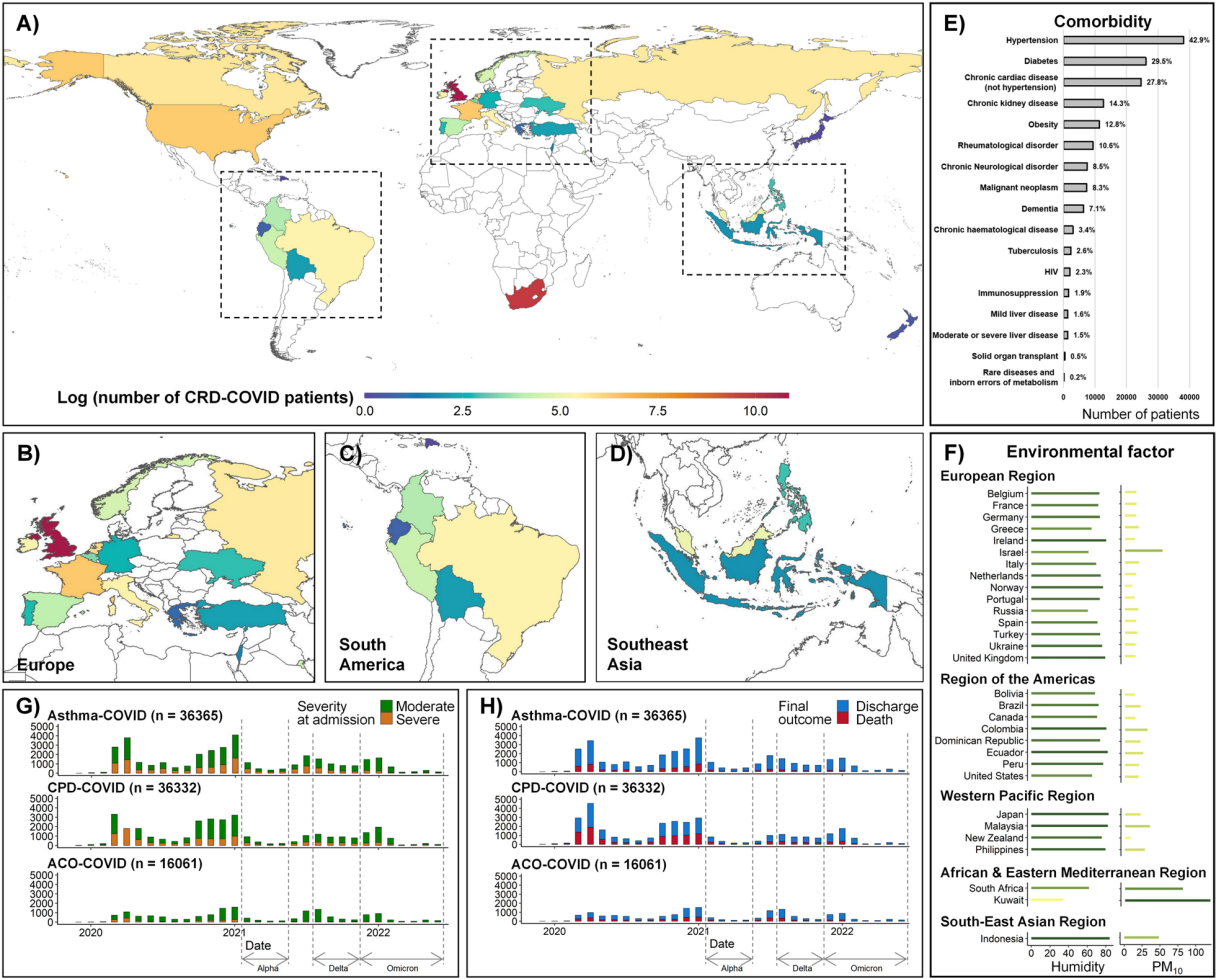
Spatiotemporal distribution and characteristics of the ISARIC CRD-COVID Cohort. **a** Global distribution of CRD-COVID patients. **b** Distribution of CRD-COVID patients in the Europe. **c** Distribution of CRD-COVID patients in the south America. **d** Distribution of CRD-COVID patients in the southeast Asia. **e** The number and proportion of other comorbidities in patients with CRD-COVID. **f** Monthly average relative humidity and PM_10_ for 30 countries. **g** Number of severe and moderate admissions per month among asthma, CPD and ACO patients from Jan 2020 to Jun 2022. **h** Number of deaths and discharges per month among asthma, CPD and ACO patients from Jan 2020 to Jun 2022

CRD-COVID patients in the ISARIC database were from 30 countries on six continents (Fig. 1a–d). CPD patients with a median age of 75 years and 56.2% males were older and had higher male proportion than asthma (median age: 59 years, 41.5% male) and ACO (median age: 67 years, 45.6% male) patients (Table 1). Comorbidities were more prevalent in CPD (57.8% CCI ≥ 2) and ACO patients (50.9% CCI ≥ 2) than in asthma patients (19.9% CCI ≥ 2). Among the 37 comorbidities, hypertension and diabetes emerged as the most common (Fig. 1e). The ranges of meteorological and air pollution conditions were similar in the asthma, CPD, and ACO cohorts and varied considerably among patients within the cohorts. Monthly average relative Humidity ranged from 16.8% to 89.7% and average monthly PM_10_ ranged from 5.4 to 164 μg/m^3^ (Fig. 1f). Infection occurred during different pandemic phases, mainly including Alpha (12.2%), Delta (16.3%), and Omicron (11.6%). The rate of severe admissions was 33.7% in the asthma cohort, 30.1% in the CPD cohort, and 15.9% in the ACO cohort (Fig. 1g). Correspondingly, 28-day mortality rates were 16.4%, 31.6%, and 25.8% in the three CRD-COVID cohorts, respectively (Fig. 1h). 31.5% of severe cases and 9.9% of moderate cases died within 28 days in asthma patients, 51.3% of severe cases and 25.2% of moderate cases died within 28 days in CPD patients, and 50.6% of severe cases and 22.9% of moderate cases died within 28 days in ACO patients.

**Table 1:** Characteristics of Asthma-, CPD- and ACO patients

Variable	N	Asthma	CPD	ACO	All
		(N = 36,365)	(N = 36,332)	(N = 16,061)	(N = 88,758)
*Indivudual factors*					
Age (median, IQR)	88,758	59.0 [45.0, 74.0]	75.0 [67.0, 82.0]	67.0 [55.0, 77.0]	69.0 [55, 79]
Male (n,%)	88,758	15,083 (41.5%)	23,746 (56.2%)	7318 (45.6%)	46,147 (48.7%)
Smoking (n,%)	40,781	6436 (35.1%)	17,445 (75.5%)	3434 (69.6%)	27,315 (58.5%)
Healthcare worker (n,%)	76,547	1567 (5.1%)	349 (0.8%)	137 (0.9%)	2053 (2.5%)
CCI (n,%)	88,758				
> 6		217 (0.6%)	1467 (3.5%)	1247 (7.8%)	2931 (3.1%)
0–1		28,907 (79.5%)	16,377 (38.8%)	6639 (41.3%)	51,923 (54.8%)
2–6		7241 (19.9%)	24,396 (57.8%)	8175 (50.9%)	39,812 (42.1%)
Hypertension (n,%)	76,273	13,808 (42.2%)	18,880 (52.2%)	7890 (52.5%)	40,578 (49.4%)
Diabetes (n,%)	83,964	9603 (27.0%)	12,318 (30.7%)	5623 (35.5%)	27,544 (30.6%)
Obesity (n,%)	59,197	5973 (22.0%)	4560 (13.8%)	1392 (18.9%)	11,925 (18.3%)
Ethnicity group (n,%)	75,914				
White		19,465 (59.0%)	28,769 (75.7%)	5840 (69.9%)	54,074 (66.1%)
Black		834 (2.5%)	432 (1.0%)	114 (1.4%)	1380 (1.7%)
Asian		2259 (6.9%)	857 (2.0%)	316 (3.8%)	3432 (4.2%)
LatinAmerican		183 (0.6%)	79 (0.2%)	6 (0.1%)	268 (0.3%)
Other		10,231 (31.0%)	10,359 (21.0%)	2078 (24.9%)	22,668 (27.7%)
*COVID-19 related factors*					
COVID-19 vaccine (n,%)	19,224	3240 (29.5%)	4134 (36.2%)	944 (39.0%)	8318 (33.1%)
Lineage (n,%)	88,758				
Other		17,796 (48.9%)	23,637 (56.0%)	6129 (38.2%)	47,562 (50.2%)
Alpha		4581 (12.6%)	4549 (10.8%)	1066 (6.6%)	10,196 (10.8%)
Beta		2418 (6.6%)	1911 (4.5%)	2371 (14.8%)	6700 (7.1%)
Gamma		22 (0.1%)	20 (0.0%)	1 (0.0%)	43 (0.0%)
Delta		6955 (19.1%)	6561 (15.5%)	3998 (24.9%)	17,514 (18.5%)
Omicron		4593 (12.6%)	5562 (13.2%)	2496 (15.5%)	12,651 (13.4%)
*Enviromental factors*					
Temperature, °C (median, IQR)	88,758	10.1 [6.4, 14.6]	10.1 [6.4, 12.7]	12.7 [8.8, 17.7]	10.1 [6.4, 14.6]
Precipitation, mm (median, IQR)	88,758	68.1 [32.8, 116.6]	61.6 [32.8, 116.6]	61.1 [26.8, 117.0]	64.6 [32.8, 116.6]
Wind speed, m/s (median, IQR)	88,758	4.18 [3.3, 4.6]	4.2 [3.8, 4.7]	3.37 [3.0, 4.3]	3.95 [3.3, 4.6]
Relative humidity, % (median, IQR)	88,758	77.6 [68.8, 84.5]	79.4 [68.8, 84.5]	70.7 [55.6, 80.3]	76.0 [68.8, 84.3]
SO_2_, μg/m^3^ (median, IQR)	88,758	2.39 [2.2, 6.0]	2.3 [2.2, 2.5]	14.9 [2.3, 32.7]	2.39 [2.2, 14.9]
NO_2_, μg/m^3^ (median, IQR)	88,758	15.6 [12.1, 18.3]	15.6 [11.7, 18.0]	16.2 [12.7, 21.7]	15.6 [12.1, 18.2]
O_3_, μg/m^3^ (median, IQR)	88,758	49.1 [41.5, 57.0]	54.9 [41.5, 57.3]	46.4 [43.0, 55.1]	49.9 [42.9, 57.0]
PM_10_, μg/m^3^ (median, IQR)	88,758	15.5 [11.8, 27.6]	15.1 [11.8, 18]	27.6 [13.9, 101.0]	16.2 [11.8, 27.6]
PM_2.5_, μg/m^3^ (median, IQR)	88,758	9.86 [7.5, 19.2]	9.4 [7.5, 12]	19.2 [9.2, 71.4]	9.86 [7.5, 19.2]

### Effect of prehospital risk factors on CRD-COVID severity and 28-day mortality

**Fig. 2:**
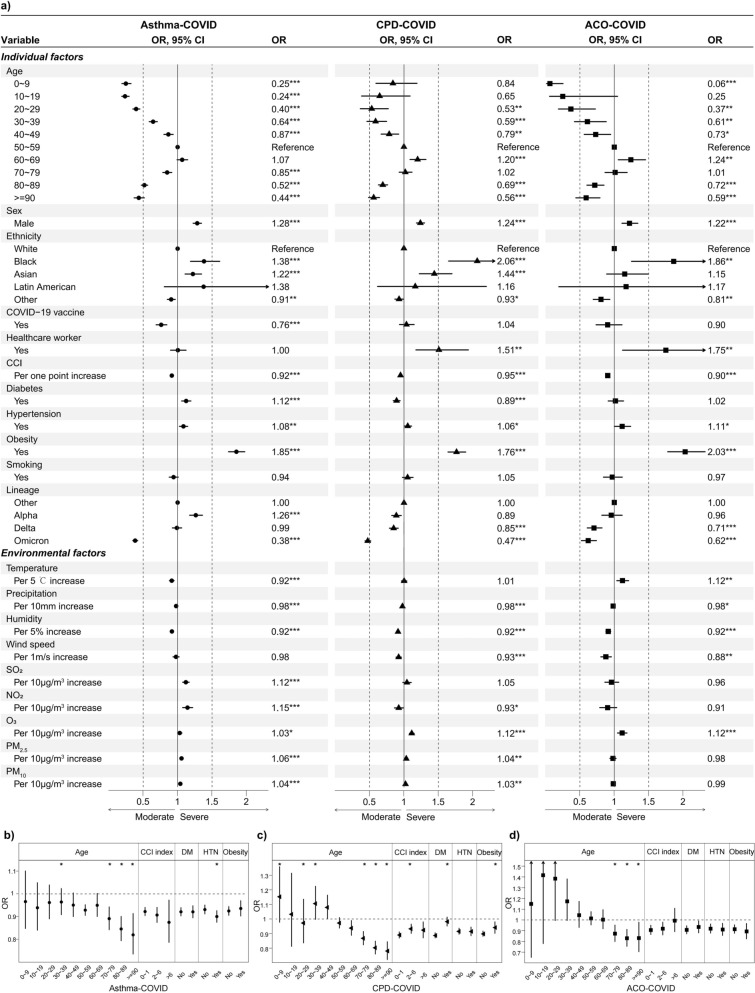
Effects and effect modification of risk factors on COVID-19 severity at admission. **a** The association between individual and environmental factors and COVID-19 severity. Statistical significance between high and low altitude: *p < 0.05; **p < 0.01; ***p < 0.001. **b** Effect modification of the association between exposure to relative humidity and COVID-19 severity in asthma patients. **c** Effect modification of the association between exposure to relative humidity and COVID-19 severity in CPD patients. **d** Effect modification of the association between exposure to relative humidity and COVID-19 severity in ACO patients. Statistically significant terms are denoted by *

Common factors that significantly impacted severe hospital admissions in asthma, CPD, and ACO patients included six individual factors: age, sex, ethnicity, CCI, hypertension, and obesity, and three environmental factors: relative humidity, precipitation, and O_3_, as well as viral variants (Fig. 2a). Notably, in CPD and ACO patients, the highest risks of severity at admission were observed in the 60–69 age group, but 50–69 years of age in asthma patients. Comorbid hypertension and obesity were associated with a higher risk of severe COVID-19, with the strongest effect especially in ACO patients (ORs: 1.11 and 2.03, both *p* < 0.05). Furthermore, other factors differed in the significance of their association with severe admissions in the three patient categories. For example, non-vaccination, low temperatures, and high SO_2_ concentrations were significantly associated with severe admissions in asthma patients, but this association was not observed in the other two categories of patients. Additionally, an 8% increase in the risk of severe admission for every 5% decrease in humidity in all CRD-COVID patients (all *P* < 0.001). Lower Humidity levels were more strongly associated with increased severity in patients over 60 (Fig. 2b-d). asthma patients with comorbid hypertension were more susceptible to severe COVID-19 under low humidity conditions (Fig. 2b). In contrast, the association between humidity and severity was weaker in CPD patients with diabetes, obesity, and moderate CCI scores (Fig. 2c).

Supplementary analysis of mortality outcomes revealed that the individual factors commonly associated with severe disease at admission among CRD-COVID patients (i.e., age, sex, ethnicity, CCI, and obesity) were also significantly associated with 28-day mortality, with the exception of hypertension (Table 2). The protective effect of vaccination extended beyond asthma patients to all groups, with unvaccinated individuals exhibiting a 32% to 46% higher risk of death. The age effect Shifted from a previously observed peak in the 50–69 age group to a continuously increasing risk with advancing age. Hypertension, previously a significant risk factor in CPD and ACO patients, became non-significant. While the direction of environmental effects remained consistent, their magnitude systematically attenuated: the mortality risk increase associated with each 5% decrease in Humidity declined from 8% to 1–2%, and the effects of air pollutants were also reduced.

**Table 2:** The association between individual and environmental factors and COVID-19 28-day

Variable	Asthma-COVID		CPD-COVID		ACO-COVID	
	HR	*P* value	HR	*P* value	HR	*P* value
Age						
0 ~ 9	0.07	< 0.001	0.14	< 0.001	0.06	< 0.001
10 ~ 19	0.11	< 0.001	0.14	0.006	0.18	0.001
20 ~ 29	0.14	< 0.001	0.57	0.013	0.35	< 0.001
30 ~ 39	0.44	< 0.001	0.71	0.014	0.61	< 0.001
40 ~ 49	0.80	< 0.001	0.67	< 0.001	0.62	< 0.001
50 ~ 59	1.00	Reference	1.00	Reference	1.00	Reference
60 ~ 69	1.78	< 0.001	1.50	< 0.001	1.44	< 0.001
70 ~ 79	2.60	< 0.001	1.95	< 0.001	1.84	< 0.001
80 ~ 89	3.53	< 0.001	2.49	< 0.001	2.49	< 0.001
>= 90	4.73	< 0.001	3.01	< 0.001	2.76	< 0.001
Sex						
Male	1.17	< 0.001	1.24	< 0.001	1.26	< 0.001
Ethnicity						
White	1.00	Reference	1.00	Reference	1.00	Reference
Black	1.23	< 0.001	1.28	0.014	1.07	0.732
Asian	1.23	< 0.001	1.17	0.026	1.22	0.059
Latin American	1.01	0.973	1.33	0.172	1.18	0.812
Other	1.36	< 0.001	1.11	< 0.001	1.19	0.001
COVID-19 vaccine						
Yes	0.54	< 0.001	0.68	< 0.001	0.57	< 0.001
Healthcare worker						
Yes	0.52	< 0.001	0.71	0.024	0.74	0.200
CCI index						
	1.10	< 0.001	1.06	< 0.001	1.05	< 0.001
Diabetes						
Yes	1.12	< 0.001	1.02	0.360	1.17	< 0.001
Hypertension						
Yes	1.13	< 0.001	0.97	0.182	1.04	0.299
Obesity						
Yes	1.42	< 0.001	1.21	< 0.001	1.17	0.010
Smoking						
Yes	1.05	0.276	1.01	0.854	1.04	0.520
Lineage						
Other	1.00	Reference	1.00	Reference	1.00	Reference
Alpha	1.14	0.003	1.02	0.515	1.19	0.007
Delta	0.94	0.179	0.82	< 0.001	1.07	0.175
Omicron	0.43	< 0.001	0.51	< 0.001	0.50	< 0.001
Temperature						
Per 5 °C	0.99	0.107	0.99	< 0.001	0.98	0.001
Precipitation						
Per 10 mm	1.00	< 0.001	1.00	0.042	1.00	0.226
Humidity						
Per 5%	0.98	< 0.001	0.99	< 0.001	0.99	0.003
Wind speed						
Per 1 m/s	0.95	0.013	0.96	0.010	0.98	0.612
SO_2_						
Per 10ug/m^3^	1.01	< 0.001	1.01	< 0.001	1.01	0.001
NO_2_						
Per 10ug/m^3^	1.04	< 0.001	1.02	< 0.001	1.02	< 0.001
O_3_						
Per 10ug/m^3^	1.00	0.414	0.99	< 0.001	0.99	< 0.001
PM_2.5_						
Per 10ug/m^3^	1.01	< 0.001	1.01	< 0.001	1.00	0.002
PM_10_						
Per 10ug/m^3^	1.00	< 0.001	1.00	< 0.001	1.00	0.001

### Contribution of each risk factor in the CRD-COVID severity and 28-day mortality prediction

**Fig. 3:**
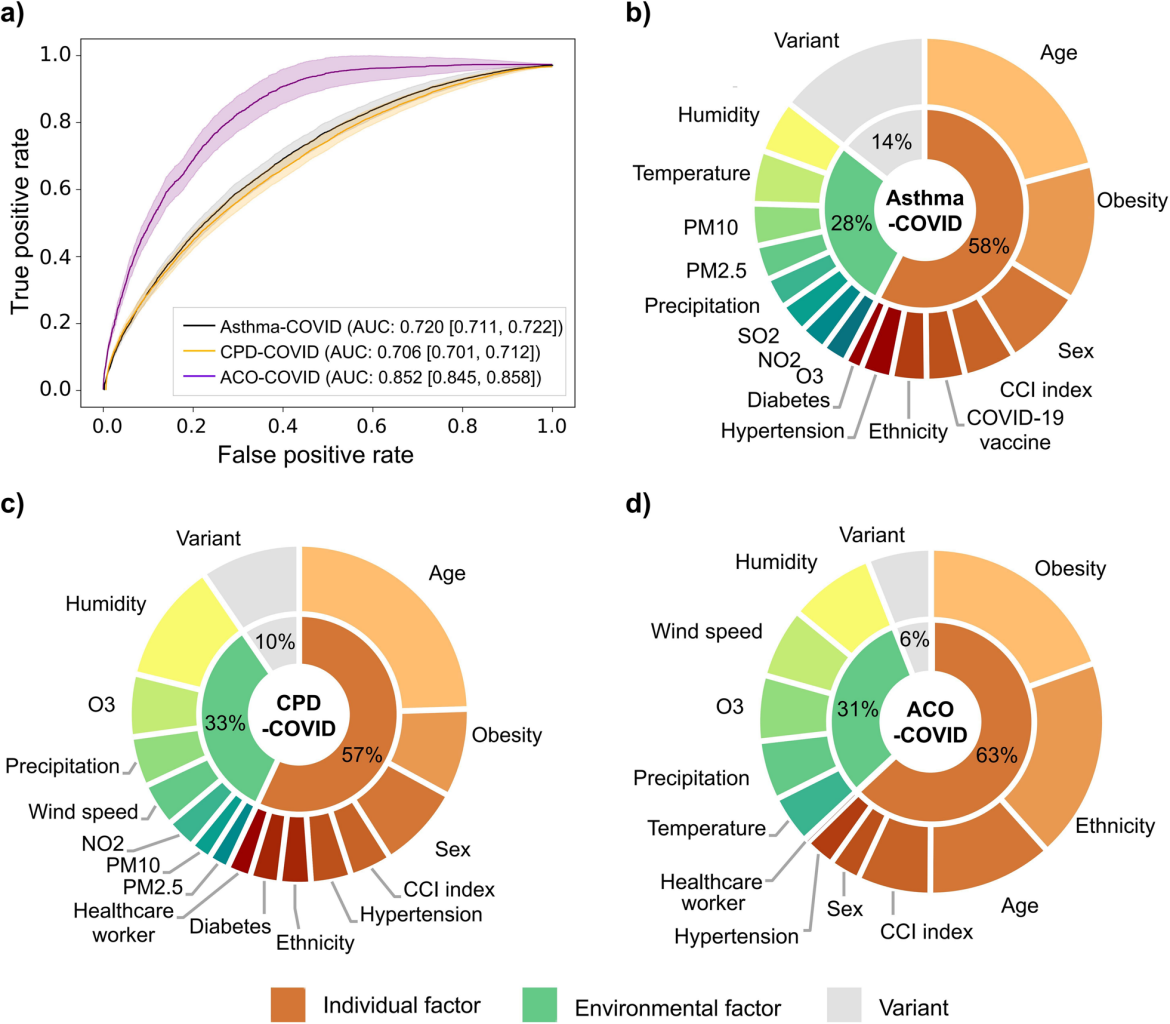
Receiver operating characteristic (ROC) curves and importance ranking of asthma, CPD and ACO models for predicting severity at admission. **a** ROC curves models predicting severity at admission in asthma, CPD and ACO models. **b** Importance ranking of individual, environmental factors and variant on admission severity in asthma patients. **c** Importance ranking of individual, environmental factors and variant on admission severity in CPD patients. **d**) Importance ranking of individual, environmental factors and variant on admission severity in ACO patients

Prehospital risk factors accurately predicted admission severity, with AUCs of 0.72, 0.70, and 0.85 for asthma, CPD, and ACO patients, respectively (Fig. 3a). SHAP importance scores showed varying proportions attributed to individual, environmental, and viral variant risks: 58%, 28%, and 14% for asthma patients; 57%, 33%, and 6% for CPD patients; and 63%, 31%, and 10% for ACO patients, respectively (Fig. 3b–d). Age, gender and comorbidities are important for all three categories of patients, with age being the most important (36.2% and 43.0% of individual factors, respectively) for asthma and CPD patients and obesity being the most important (31.0%) for ACO patients. Environmental factor impacts differed by disease: Relative humidity was more important CPD patients (34.6%) compared to others (18.0% and 26.2%), while PM_10_ concentrations impact asthma patients (14.2%) more than CPD patients (6.1%) (Supplementary table S2).

Pre-admission risk factors also effectively predicted 28-day mortality, with AUCs of 0.775, 0.685, and 0.703 for asthma, CPD, and ACO patients, respectively (supplementary figure S1). In contrast to predictors of admission severity, individual-level factors remained the primary contributors to 28-day mortality across all patient groups—accounting for 74%, 68%, and 63% in asthma, CPD, and ACO patients, respectively (supplementary table S3). Age, obesity, and CCI index continued to play dominant roles. Notably, COVID-19 vaccination showed greater contribution to mortality than to admission severity, ranking as the fourth most important factor in asthma and CPD patients, and fifth in ACO patients. Meanwhile, environmental factors showed reduced influence on mortality compared to admission severity, contributing only 16%, 20%, and 16% across the three groups.

### Prehospital risk factors-driven clusters in CRD-COVID patients

**Fig. 4:**
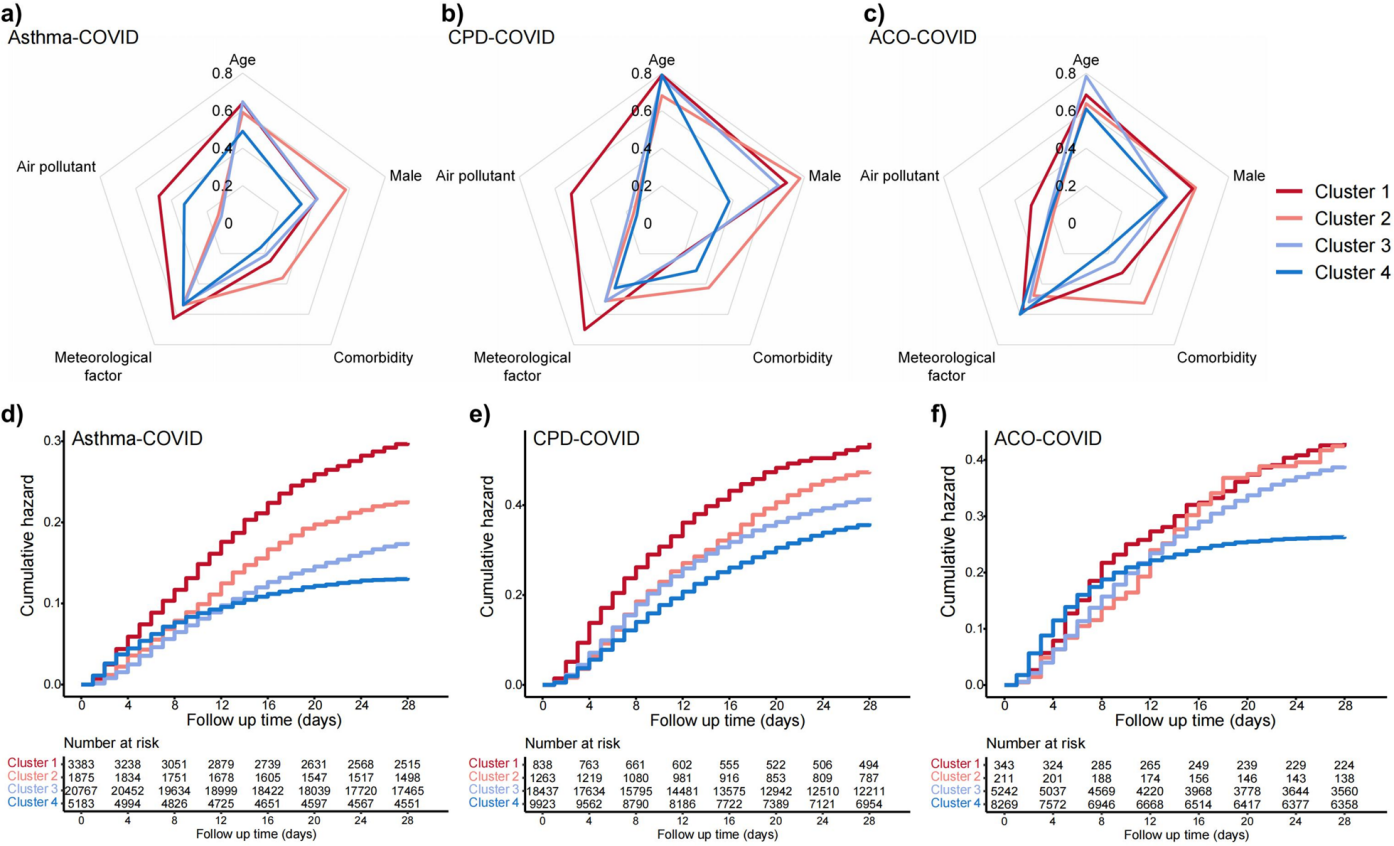
Characteristics and survival curves of four clusters in asthma, CPD and ACO patients **a** Radar charts of individual and environmental characteristics of the four clusters in asthma patients. **b** Radar charts of individual and environmental characteristics of the four clusters in CPD patients. **c** Radar charts of individual and environmental characteristics of the four clusters in ACO patients. **d** Kaplan–Meier survival curves for four clusters in asthma patients. **e** Kaplan–Meier survival curves for four clusters in CPD patients. **f** Kaplan–Meier survival curves for four clusters in ACO patients

Patients with asthma, CPD, and ACO were clustered into four subgroups (Clusters 1–4) based on prehospital risk factors and predicted severity at admission (supplementary figure S2). Radar charts illustrated differences among the clusters in five main risks: age, sex, comorbidities, meteorological conditions, and air pollution (Fig. 4a–c). The characteristics of the four clusters of asthma and CPD patients were similar (Fig. 4a, b): Cluster 1 showed the highest mortality rates (74.3% for asthma and 58.9% for CPD), and characterised by high level of meteorological risks and air pollution such as low humidity (mean: 55.5% for asthma and 54.4% for CPD) and high PM_10_ concentrations (mean: 106 μg/m^3^ for asthma and 119 μg/m^3^ for CPD), along with moderate individual risk such as older age (mean: 60.1 for asthma and 74.2 years for CPD); Cluster 2 showed high individual risks with significantly higher rates of obesity (75.2% for asthma and 62.0% for CPD) and proportion of males (56.9% for asthma and for 77.1% CPD). Differently, Cluster 4 had the lowest individual risk in asthma patients and the lowest environmental risk in CPD patients. For ACO patients, all clusters showed high individual risk, with Cluster 2 showing a high prevalence of obesity (83.9%) and Cluster 3 having a high median age (73.2 years) (Fig. 4c). Cluster 4 has the lowest individual risks, although it has adverse meteorological conditions such as the lowest relative humidity (60.6%). Detailed differences in risk factors between clusters are in the supplementary Fig. 3–5.

### Survival analysis and prehospital risk stratification in CRD-COVID patients

The 28-day mortality rates decreased sequentially from Cluster 1 to Cluster 4. In asthma, CPD, and ACO patients, mortality rates were 25.7%, 41.1%, and 34.7% for Cluster 1, and 12.2%, 29.9%, and 23.1% for Cluster 4, respectively. There was a significant difference in mortality between Cluster 1 and 4 in asthmaand CPD patients, with Cluster 1 facing 2.23 and 1.55 times higher risk of death within 28 days than Cluster 4, respectively (both *p* < 0.001) (Fig. 4d, e). In the ACO patients, only Cluster 4 showed a significantly lower mortality risk compared to the rest, with Clusters 1–3 facing 1.38–1.54 times higher mortality risks (all *p* < 0.001) (Fig. 4f).

### Sensitive analysis

The findings remained robust after excluding early pandemic data from the first quarter of 2020: effect directions and statistical significance of nearly all key variables were consistent (Supplementary Tables 6–8). The nonlinear effect of age persisted across all CRD-COVID groups. Key clinical factors, including sex, vaccination status, obesity, and diabetes, showed stable effects with minimal changes in odds ratios. Although hypertension became non-significant across all groups, its effect direction remained unchanged. Among environmental variables, relative humidity remained significant (*p* < 0.05). All air pollutants remained significant except for NO_2_ in CPD patients. Variant-related risks were consistent, with Omicron associated with lower risk (*p* < 0.001) and Alpha with higher risk (*p* < 0.001). Additionally, model performance in predicting disease severity remained robust after exclusion of early pandemic data, with AUCs of 0.722 (95% CI 0.713–0.724) for asthma-COVID, 0.704 (95% CI 0.698–0.709) for CPD-COVID, and 0.844 (95% CI 0.837–0.852) for ACO-COVID patients (supplementary figure S8).

## Discussion

This study clarifies the important role of prehospital risk factors in the prediction of severity and prognosis as well as in the risk stratification of asthma, CPD, and ACO patients. We combined traditional statistical models and advanced machine learning algorithms to fully explore real-world data in the pandemic. Our analysis found among CRD-COVID patient categories, same factors such as individual factors (age, sex, comorbidities), and environmental factors (humidity, precipitation, O_3_) significantly impacted admission severity with varied association strengths, while vaccination, temperature, and SO_2_ only showed significance in certain patient category. Particularly noteworthy were differences in the contribution of prehospital risk factors to the prediction of severity across disease groups. Furthermore, there were significant differences in survival between subgroups driven by prehospital risk factors, particularly in the asthma and CPD groups. Interestingly, the subgroup characterised by high environmental risk and moderate individual risk had the lowest survival rates compared to the subgroup characterised only by high individual risk. These findings offered valuable insights into early risk assessment and CRDs management base on prehospital risk factors.

Our study showed a non-linear relationship between age and severity among CRD-COVID patients. Specifically, asthma patients were at high risk at an earlier age compared to CPD and ACO patients. This may be due to asthma patients having the disease at a younger age, where prolonged and repeated inflammatory responses impair airway development and reduce lung function [27, 28]. Surprisingly, high CCI scores were not associated with severe admission, considering that patients with more comorbidities may have sought medical care more promptly after COVID-19 infection [29]. In line with existing studies based on general population, our findings showed that males, individuals comorbid with hypertension and diabetes, the unvaccinated were more likely to suffer severe COVID-19 [30, 31]. Although asthma is more common in female patients, male asthma patients were more likely to develop more severe outcomes of COVID-19 infection. Furthermore, we observed significant differences in disease severity among patients of different racial and occupational backgrounds. Among the three groups of COVID-19 patients, Black patients showed significantly more severe conditions at admission compared to White patients (though with wide confidence intervals). This finding helps explain the worse clinical outcomes observed for Black patients in other studies, suggesting that this health disparity already existed at the time of admission [32–34]. Among patients with CPD and ACO, healthcare workers had more severe conditions at admission than non-healthcare workers, which may be attributed to higher viral load exposure due to occupational risks [35]. Since the patients included in this study were from different periods of SARS-CoV-2 variant prevalence, we adjusted for the influence of variants in our analysis. The results showed that, consistent with other studies, the Omicron variant was associated with less severe disease, a pattern that was confirmed in patients with asthma, CPD, and ACO [36, 37]. These findings further enrich our understanding of the clinical characteristics of CRD-COVID patients and provide important evidence for risk assessment.

Environmental factors, especially those related to low Humidity, also played an important role in disease severity. Our study included CRD-COVID patients from 30 countries across six continents, living in environments with relative Humidity ranging from 17 to 90%. This global coverage allowed us to capture regional differences that single-site studies may miss. While previous research has suggested that increased humidity may worsen asthma symptoms by promoting indoor allergens such as mold and dust mites, our focus on patients with concurrent COVID-19 infection suggests a different mechanism may be at play. Low humidity can compromise the respiratory immune barrier by impairing mucociliary clearance and reducing innate antiviral defenses, thereby increasing vulnerability to severe viral infections like COVID-19 [38]. Several large-scale ecological studies have also supported this link between low humidity and worse outcomes in both COVID-19 and asthma patients [39, 40].

Moreover, our study highlighted groups that are more sensitive to adverse environments by analysing the interaction of individuals with environmental factors. The elderly are most vulnerable to low humidity among CRD-COVID patients, and asthma patients with comorbid hypertension were more sensitive to low humidity. Several other studies have also reported the vulnerability of patients with chronic diseases such as coronary heart disease, lung cancer, and dementia, whose COVID-19 severity is more susceptible to environmental factors [41, 42]. Overall, these insights clarified the impact of prehospital individual and environmental factors on the severity of CRD-COVID patients, providing information for tailored risk assessment for this vulnerable population.

Although our study primarily focused on disease severity at admission, the supplementary analysis of 28-day mortality largely confirmed the prehospital risk factors we identified—particularly age, comorbid obesity, and CCI score. Notably, the protective effect of vaccination contributed more significantly to mortality prediction than to admission severity, further underscoring the importance of vaccination across all CRD patient groups. In contrast, hypertension, while a significant predictor of severity, was no longer associated with mortality risk, possibly reflecting improved in-hospital management. The impact of environmental exposures was markedly attenuated in the mortality models, suggesting that these factors primarily influence the early course of disease. This attenuation may also reflect the confounding effects of in-hospital interventions and other factors in mortality risk models, which could obscure the direct influence of environmental exposures. By comparison, such external exposures are more readily detectable and quantifiable in analyses of admission severity. Overall, our findings highlight the dominant role of individual-level prehospital risk factors in shaping both early severity and subsequent mortality in CRD-COVID patients, while also illustrating how these influences may vary by disease stage and underlying respiratory condition.

Our study showed different contributions of prehospital risk factors in predicting severity among patients with asthma, CPD, and ACO. Individual factors, particularly age and obesity, have an important impact on the admission severity. Age was the most important individual risk factor for asthma and CPD patients, whereas obesity was the most important individual risk for ACO patients. Comorbidities such as diabetes, hypertension, and CCI index also play an important role in predicting severity, and are most important in ACO patients. Moreover, our study underscored the importance of environmental risks in predicting severe COVID-19 outcomes among CRD patients. Meteorological factors including relative humidity, temperature, precipitation, and wind speed mainly contributed to the severity of CPD and ACO patients, and air pollutants such as PM_10_ and SO_2_ contributed the most to asthma patients [43]. Notably, humidity was the most important environmental factor in all three categories of patients, particularly for the patients with CPD. The differential effects of various air pollutants suggested the need for tailored pollution exposure management strategies for different patient groups. Our findings offered valuable insights into quantifying risks and prioritising interventions to optimise CRD management.

We applied disease-specific prehospital risk factors to stratify risk in asthma, CPD, and ACO patients, introducing a novel approach rather than previous methods based on medical interventions and laboratory test indicators [44–46]. Contrary to conventional understanding, our findings challenge the notion that patients with the highest individual risk face the highest risk of death. We observed that patients exposed to the highest environmental risk, along with moderate individual risks but limited comorbidities showed the lowest 28-day survival among asthma and CPD patients. This underscores the critical role of environmental factors, such as air pollution and humidity levels, in shaping COVID-19 outcomes. Therefore, populations exposed to high environmental risks may require more comprehensive interventions and early prevention strategies, including improving environmental conditions, increasing access to healthcare services, and promoting healthy lifestyles. In addition, our analysis also showed that among patients with moderate conditions at admission, higher environmental risks did not necessarily lead to worse final outcomes, while the effects of individual risk factors were more important. Notably, among ACO patients, those with lower individual risks had the highest survival rates, even though they were exposed to relatively high environmental risks. This highlighted the importance of individual risk assessment and management in ACO patients, as their survival depended more on individual risk levels. Overall, our study underscored the significance of integrating both environmental and individual risk factors in risk stratification and management strategies for CRD-COVID patients.

Our analysis had several limitations. Firstly, we classified disease severity based only on respiratory status at hospital admission, which does not reflect later clinical deterioration. As a result, the true peak severity in some patients may have been underestimated. Secondly, our dataset lacked baseline measures of lung disease severity (e.g., FEV1, DLCO, GOLD stage), as these were not available in the ISARIC registry. Since such factors may influence disease progression, their absence could confound our results. Future studies should collect this information to enhance risk stratification. Thirdly, comorbidities were defined only based on case report forms, limiting the assessment of comorbidity severity. Forthly, This study lacked detailed vaccination information, such as vaccine type, number of doses, and timing. The binary “vaccinated” variable may include individuals with waning immunity or incomplete vaccination, potentially underestimating the protective effect of vaccines. Additionally, data on pre-hospital treatments (e.g., antivirals, corticosteroids) were unavailable, which may affect the assessment of mortality risk in the survival analysis. Nevertheless, since the primary focus of this study was disease severity at admission, the absence of in-hospital treatment data likely had limited impact on the main findings. Lastly, our study lacked patient-level residential location and daily-resolution data, which limited the precision in identifying environmental risk factors. Future research incorporating high-resolution health outcomes and environmental exposures would be better suited to accurately capture the delayed dynamics of environmental health effects. These limitations underscore the need for further research in these areas. Moreover, given the study period and population, caution should be exercised when generalizing these findings to later phases of the pandemic marked by new variants and widespread vaccination.

## Conclusions

This study highlighted the importance of integrating prehospital individual, environmental, and viral factors to understand their impact on CRD-COVID severity. We demonstrated the crucial role of prehospital risk factors in severity prediction, disease progression assessment, and risk stratification. Through analysis and comparison across three categories of diseases, we identified heterogeneous risks, providing insights into emerging health challenges. Moreover, integrating traditional statistical models with advanced machine learning enhanced the prediction accuracy, facilitating precise patient stratification. Overall, this study provides a basis for the development of targeted early prevention strategies and a paradigm for the management of acute respiratory infectious disease pandemics in CRD patients.

## Supplementary Information

Supplementary file 1.

## Data Availability

The ISARIC COVID-19 datasets used in this study are available in the Infectious Diseases Data Observatory (IDDO, www.iddo.org) Data will be shared after approval of the proposal and with a signed data access agreement.
