# Supplementary material for: Chronic respiratory diseases risk during the COVID-19 pandemic: an integrated modelling approach based on hospital records across 30 countries
Source: Popul Health Metr. 2025 Nov 6;23:61. doi: 10.1186/s12963-025-00412-x (PMC12593867; doi:10.1186/s12963-025-00412-x)
Supplement: Supplementary file 1 [file 12963_2025_412_MOESM1_ESM.docx]

**Online Supplement for Chronic respiratory diseases risk during the COVID-19 pandemic: an integrated modelling approach based on hospital records across 30 countries**

**Methods**

This study is a secondary analysis of the ISARIC COVID-19 dataset, which is one of the largest international databases of prospectively collected clinical data on people hospitalized with COVID-19. Investigators from 60 countries collected prospective data on patients with clinically suspected or laboratory-confirmed COVID-19 infection using the ISARIC Case Report Form (CRF) built on Research Electronic Data Capture (REDCap, version 8.11.11, Vanderbilt University, Nashville, Tenn.) hosted by the University of Oxford. The data collection methods and characteristics have been described elsewhere [12]. The ISARIC-WHO Clinical Characterization Protocol received approval from the WHO Ethics Review Committee (RPC571 and RPC572). Each participating country and site obtained local ethics approval in accordance with their specific requirements. Furthermore, this work is licensed under the Creative Commons Attribution 4.0 International License by IDDO on behalf of the University of Oxford. This study was ethically exempted by the Human Research Ethics Committee of Tsinghua University (Project No. 20220163).

**Ethnicity**

The ethnicities included in our analyses included White, Black, Asian, and Latin American, and other ethnicities with smaller sample sizes, such as Arab and Mixed, were classified as Other.

**Charlson Comorbidity Index (CCI)**

Charlson Comorbidity Index (CCI) was calculated with an ad hoc modified formula, i.e. congestive heart failure, peripheral vascular disease, chronic pulmonary disease, dementia, peptic ulcer disease, mild liver disease, diabetes, and rheumatism gave one point each, history of chronic haematological diseases, chronic kidney disease, and malignant neoplasms gave two points each, history of moderate and severe liver disease gave three points and acquired immunodeficiency syndrome (AIDS) gave six points. The CCI is classified as mild (0-1), moderate (2-6) or severe (>6) in our study.

**SARS-CoV-2 variants**

SARS-CoV-2 lineage data from January 2020 to July 2022 were obtained from an integrated global SARS-CoV-2 database, the China National Center for Bioinformation (CNCB), which includes data from the Global Initiative on Sharing All Influenza Data (GISAID), NCBI GenBank, National Genomics Data Center (NGDC), National Microbiology Data Center (NMDC), and China National GeneBank (CNGB). This database also provides variants identified from these sequences. Variant included in the analysis as a multicategorical variable.

**Environmental data and its pre-processing**

Meteorological factors including temperature, precipitation, relative humidity, and wind speed were extracted from ERA5 monthly averaged data with a spatial resolution of 0.25° x 0.25° [18,19]. Air pollutants included SO2, NO2, O3, PM10, PM2.5 from CAMS global reanalysis (EAC4) monthly averaged with a spatial resolution of 0.75°x0.75° [20]. All environmental data are pre-processed with population weighting. For each country, we divided the sum of values of environmental indicators at the grid cell by population at the grid cell within each country, divided by the sum of population per grid cells of each country. Population data was from the Gridded Population of the World for 2020, with a spatial resolution of 30 arc-second [21,22].

**Recursive Feature Elimination (RFE) algorithm**

The RFE strategy used all the important factors to train a supervised model, and then evaluates the factors based on their importance in the model. In each iteration, only one factor with the least importance to the model is eliminated, and the model fit in each iteration is compared using AUC to finally select the combination of factors that makes the best model fit.

**Hyperparameter Tuning**

The best combination of hyperparameter values was selected using a five-fold cross-validation grid search. The tuned parameters included the learning rate (0.01, 0.05, 0.1, 0.5, 1) and the maximum depth of the tree (from 1 to 10, with a spacing of 1), and the minimum number of samples retained per leaf node (10, 100, 1000). The objective function was set to "bernoulli_logit" because our dependent variable of interest is a binary categorical variable. The training process was stopped when the performance of the validation dataset did not improve after further training iterations. The dataset was split into three parts: 60% for training, 20% for validation, and 20% for testing.

**Results**

**Characteristics of the clusters in Asthma-, CPD-, and ACO-COVID patients**

Patients with Asthma-, CPD-, and ACO-COVID were respectively clustered into four subgroups based on predicted severity at admission and disease-specific predictors. In detail, for Asthma-COVID patients, Cluster 1 was characterized by high air pollution and meteorological risks including low humidity (55.5%) and high PM_10_ levels (106 μg/m^3^), coupled with moderate individual risks such as older age (mean: 60.1) (supplementary figure S2). Both Cluster 2 and Cluster 3 showed relatively high individual risks, but Cluster 2 had a significantly higher obesity rate (75.2%) and proportion of males (56.9%) than Cluster 3. Cluster 4 had the lowest individual risk, characterised by the lowest age (46.2), the lowest proportion of males (31.6%) and the lowest rates of obesity (4.6%), hypertension (34.7%) and diabetes (21.4%). In the CPD-COVID patients, similar to Asthma-COVID patients, Cluster 1 characterized by low humidity (54.4%) and high air pollution. Cluster 2, characterized by the high individual risks, had more males (77.1%) and high rates of obesity (62.0%) and diabetes (39.9%), but lower air pollution and meteorological risks (supplementary figure S3). Although patients in Cluster 4 were very old (73.7 years), their remaining risks were all the lowest. For ACO-COVID patients, Cluster 1-3 all showed high individual risk, with Cluster 2 having more obese patients (83.9%) and Cluster 3 having more elderly patients (73.2 years) (supplementary figure S4). Cluster 4 has the lowest individual risks, although it has adverse meteorological conditions such as the lowest relative humidity (60.6%).

**​**

***Supplementary figure***

**
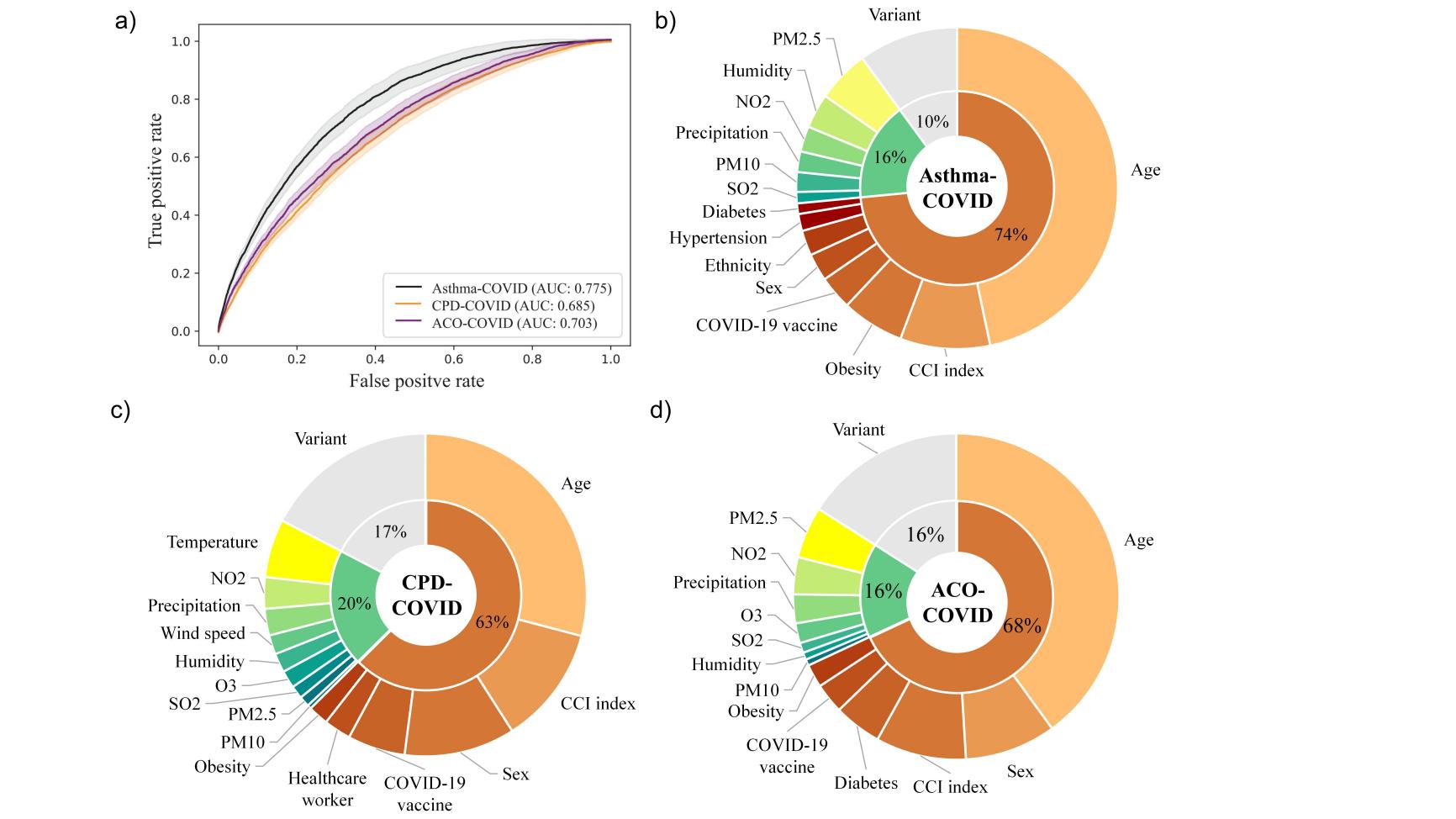
**

Supplementary figure 1. Receiver operating characteristic (ROC) curves and importance ranking of asthma, CPD and ACO models for predicting 28-day mortality. a) ROC curves models predicting 28-day mortality in asthma, CPD and ACO models. b) Importance ranking of individual, environmental factors and variant on 28-day mortality in asthma patients. c) Importance ranking of individual, environmental factors and variant on 28-day mortality in CPD patients. d) Importance ranking of individual, environmental factors and variant on 28-day mortality in ACO patients.

***
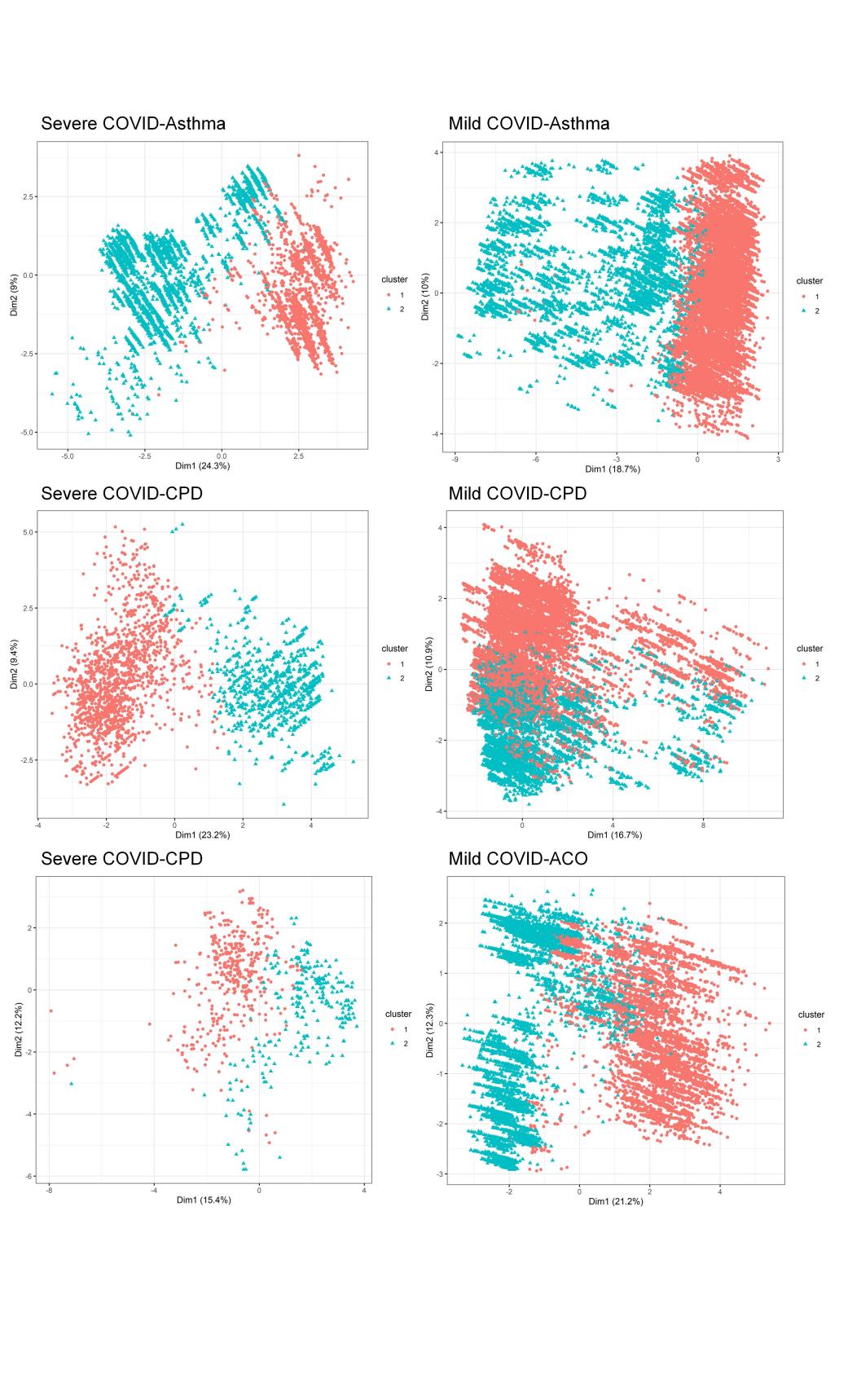
***

Supplementary figure 2. Clustering results based on predicted severity at admission and disease-specific predictors in Asthma-, CPD- and ACO-COVID patients.


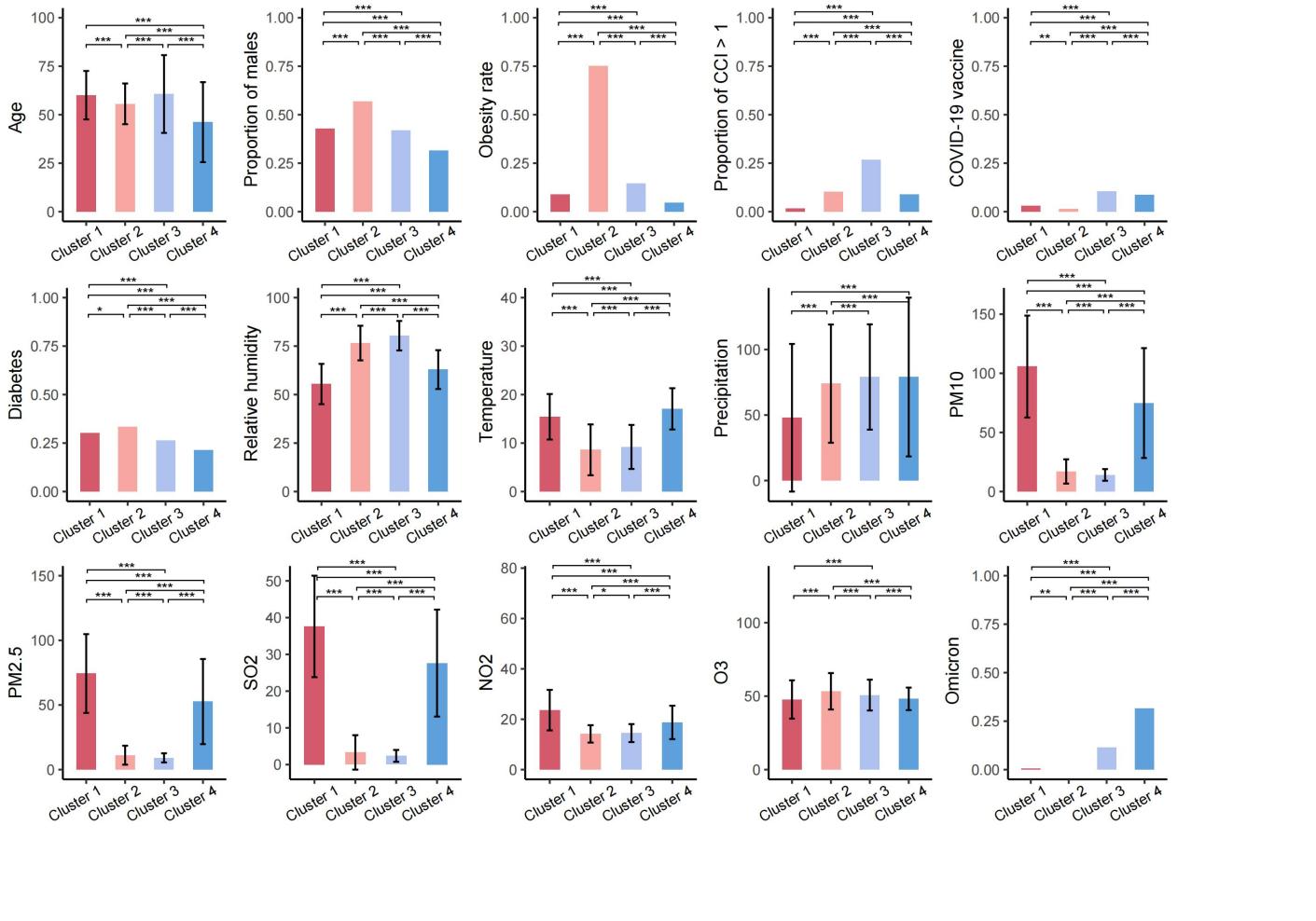


Supplementary figure 3. Differences across four clusters of important individual, environmental and viral variant factors in Asthma-COVID patients.


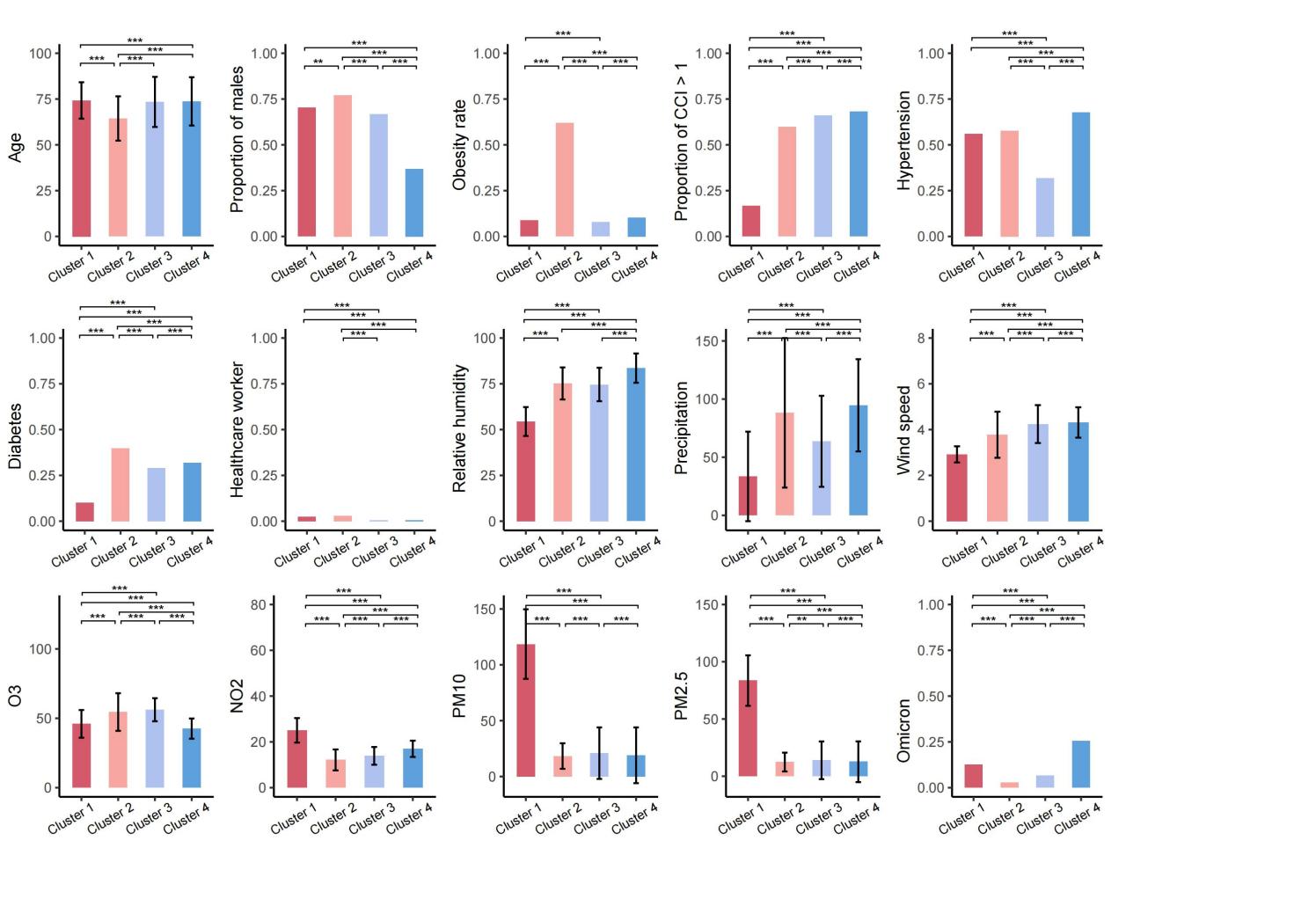


Supplementary figure 4. Differences across four clusters of important individual, environmental and viral variant factors in CPD-COVID patients.


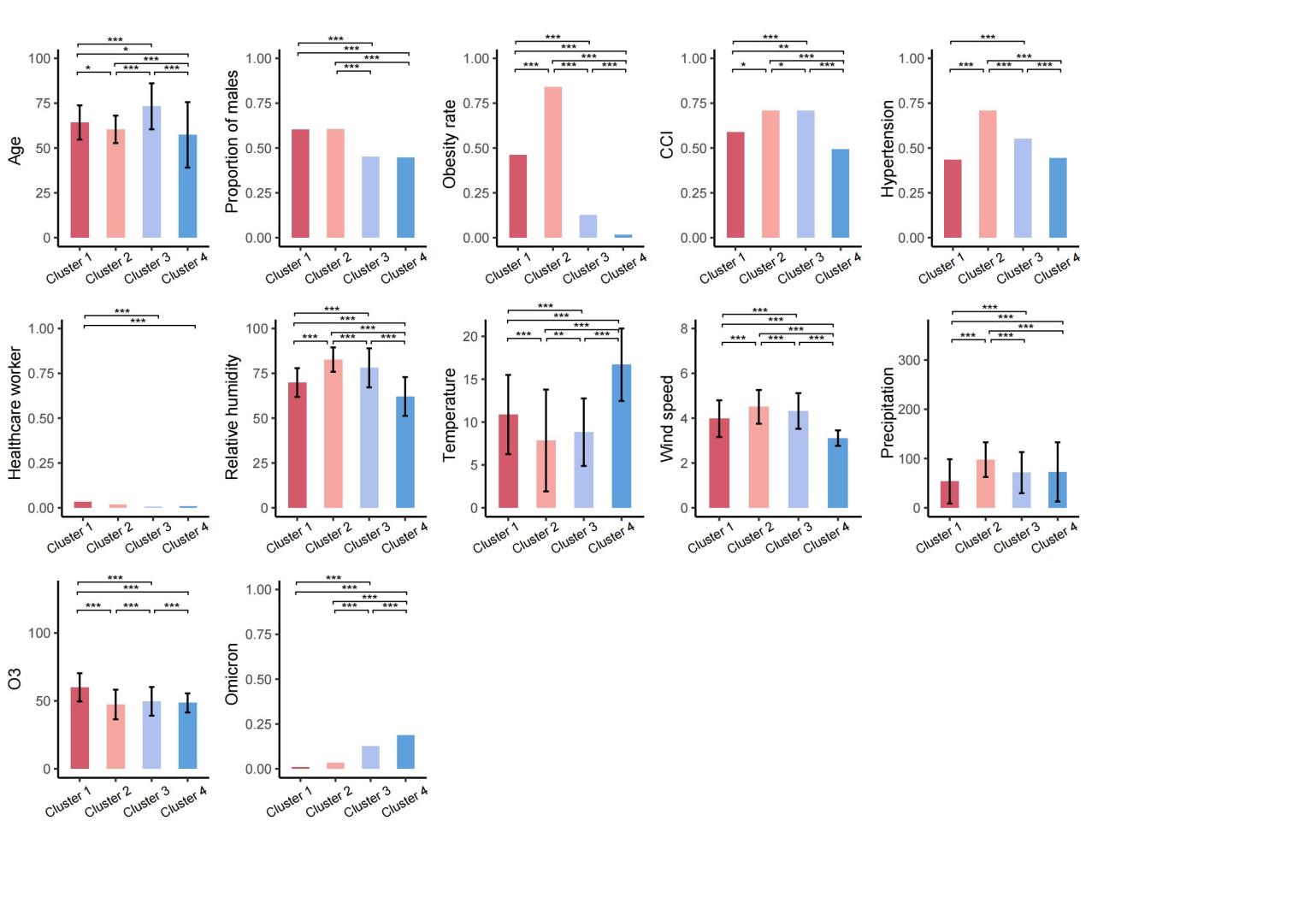


Supplementary figure 5. Differences across four clusters of important individual, environmental and viral variant factors in ACO-COVID patients.


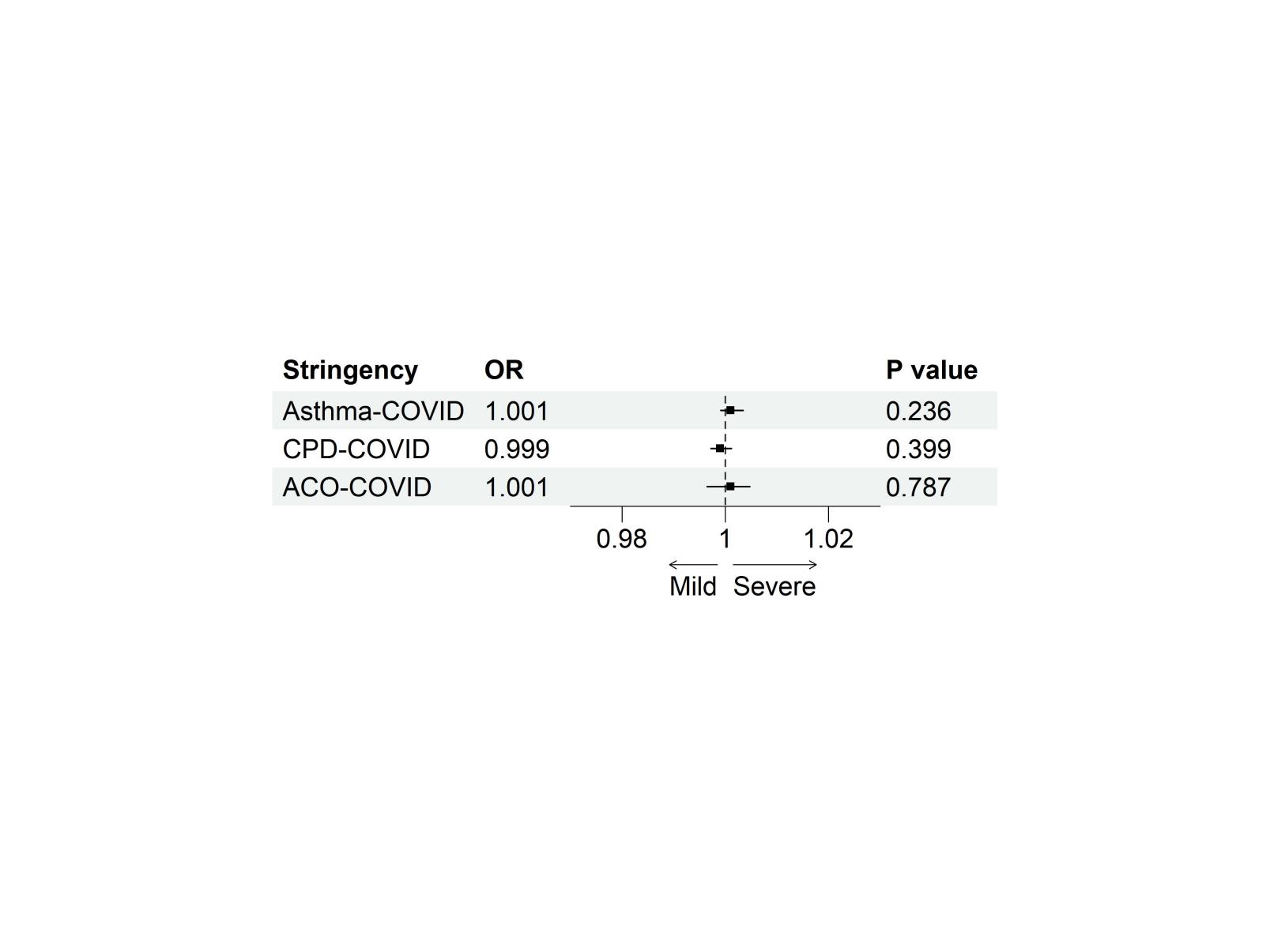


Supplementary figure 6. Effects of the stringency of COVID-19 public health and social measures on COVID-19 severity at admission in asthma-, CPD- and ACO-COVID patients, adjusting all individual factors including age, sex, ethnicity, COVID-19 vaccination, healthcare worker, CCI index, diabetes, hypertension, obesity, smoking, and lineage.


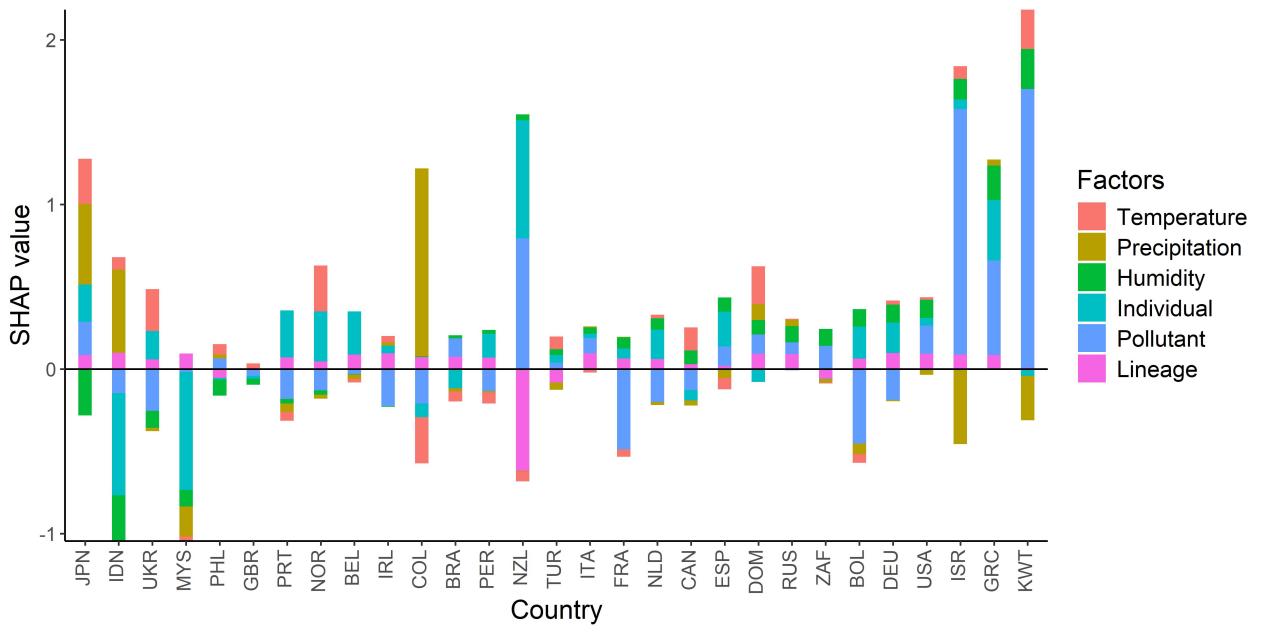


Supplementary figure 7. The protection and risk contributions of the risk factors for CRD-COVID severity in each country. SHAP values above 0 are regarded as risk effects and below 0 as protective effects.


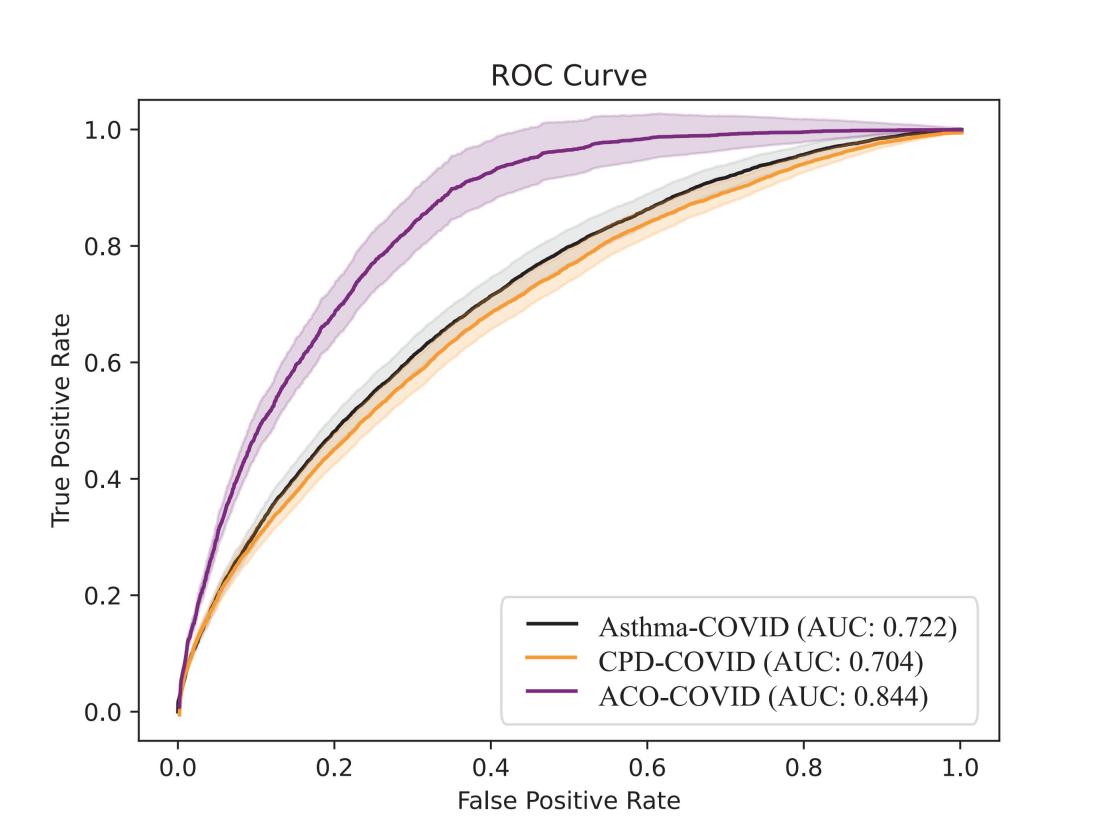


Supplementary figure 8. ROC curves of asthma, CPD and ACO models in sensitive analysis.

***Supplementary table***

Supplementary table 1. Variable selection of Asthma-, CPD- and ACO-COVID models

| Asthma-COVID model |  |
| --- | --- |
| **Variable** | **AUC** |
| Age', 'Sex', 'COVID-19 vaccine', 'CCI', 'Diabetes', 'Hypertension', 'Obesity', 'Temperature', 'Precipitation', 'Relative humidity', 'SO2', 'NO2', 'O3', 'PM10', 'PM2.5', 'Ethnic_Asian', 'Ethnic_Black', 'Ethnic_LatinAmerican', 'Ethnic_White', 'Ethnic_Other', 'Lineage_Alpha', 'Lineage_Beta', 'Lineage_Delta', 'Lineage_Gamma', 'Lineage_Omicron', 'Lineage_Other' | 0.702623941 |
| Age', 'Sex', 'COVID-19 vaccine', 'CCI', 'Diabetes', 'Hypertension', 'Obesity', 'Temperature', 'Precipitation', 'Relative humidity', 'SO2', 'NO2', 'O3', 'PM10', 'PM2.5', 'Ethnic_Asian', 'Ethnic_Black', 'Ethnic_White', 'Ethnic_Other', 'Lineage_Delta', 'Lineage_Omicron', 'Lineage_Other' | 0.702802494 |
| Age', 'Sex', 'COVID-19 vaccine', 'CCI', 'Diabetes', 'Hypertension', 'Obesity', 'Temperature', 'Precipitation', 'Relative humidity', 'SO2', 'NO2', 'O3', 'PM10', 'PM2.5', 'Ethnic_Asian', 'Ethnic_Black', 'Ethnic_Other', 'Lineage_Delta', 'Lineage_Omicron', 'Lineage_Other' | 0.702522253 |
| Age', 'Sex', 'COVID-19 vaccine', 'CCI', 'Hypertension', 'Obesity', 'Temperature', 'Precipitation', 'Relative humidity', 'SO2', 'NO2', 'O3', 'PM10', 'PM2.5', 'Ethnic_Asian', 'Ethnic_Black', 'Ethnic_Other', 'Lineage_Delta', 'Lineage_Omicron', 'Lineage_Other' | 0.702023743 |
| Age', 'Sex', 'COVID-19 vaccine', 'CCI', 'Diabetes', 'Hypertension', 'Obesity', 'Temperature', 'Precipitation', 'Relative humidity', 'SO2', 'NO2', 'O3', 'PM10', 'PM2.5', 'Ethnic_Asian', 'Ethnic_Black', 'Ethnic_Other', 'Lineage_Omicron', 'Lineage_Other' | 0.701802349 |

| CPD-COVID model |  |
| --- | --- |
| **Variable** | **AUC** |
| Age', 'Sex', 'Healthcare worker', 'CCI', 'Diabetes', 'Hypertension', 'Obesity', 'Precipitation', 'Relative humidity', 'Wind speed', 'NO2', 'O3', 'PM10', 'PM2.5', 'Ethnic_Asian', 'Ethnic_Black', 'Ethnic_LatinAmerican', 'Ethnic_White', 'Ethnic_Other', 'Lineage_Alpha', 'Lineage_Beta', 'Lineage_Delta', 'Lineage_Gamma', 'Lineage_Omicron', 'Lineage_Other' | 0.664036397 |
| Age', 'Sex', 'Healthcare worker', 'CCI', 'Diabetes', 'Hypertension', 'Obesity', 'Precipitation', 'Relative humidity', 'Wind speed', 'NO2', 'O3', 'PM10', 'PM2.5', 'Ethnic_Asian', 'Ethnic_Black', 'Ethnic_White', 'Ethnic_Other', 'Lineage_Alpha', 'Lineage_Beta', 'Lineage_Omicron', 'Lineage_Other' | 0.661004683 |
| Age', 'Sex', 'Healthcare worker', 'CCI', 'Diabetes', 'Hypertension', 'Obesity', 'Precipitation', 'Relative humidity', 'Wind speed', 'NO2', 'O3', 'PM10', 'PM2.5', 'Ethnic_Asian', 'Ethnic_Black', 'Ethnic_White', 'Ethnic_Other', 'Lineage_Beta', 'Lineage_Omicron', 'Lineage_Other' | 0.66100765 |
| Age', 'Sex', 'Healthcare worker', 'CCI', 'Diabetes', 'Hypertension', 'Obesity', 'Precipitation', 'Relative humidity', 'Wind speed', 'NO2', 'O3', 'PM10', 'PM2.5', 'Ethnic_Asian', 'Ethnic_Black', 'Ethnic_White', 'Ethnic_Other', 'Lineage_Omicron', 'Lineage_Other' | 0.663999362 |
| Age', 'Sex', 'Healthcare worker', 'CCI', 'Diabetes', 'Hypertension', 'Obesity', 'Precipitation', 'Relative humidity', 'Wind speed', 'NO2', 'O3', 'PM10', 'PM2.5', 'Ethnic_Asian', 'Ethnic_Black', 'Ethnic_White', 'Ethnic_Other', 'Lineage_Omicron' | 0.662347632 |
| Age', 'Sex', 'Healthcare worker', 'CCI', 'Diabetes', 'Hypertension', 'Obesity', 'Precipitation', 'Relative humidity', 'Wind speed', 'NO2', 'O3', 'PM10', 'PM2.5', 'Ethnic_Black', 'Ethnic_White', 'Ethnic_Other', 'Lineage_Omicron' | 0.661610918 |
| Age', 'Sex', 'Healthcare worker', 'CCI', 'Diabetes', 'Hypertension', 'Obesity', 'Precipitation', 'Relative humidity', 'Wind speed', 'NO2', 'O3', 'PM10', 'PM2.5', 'Ethnic_White', 'Ethnic_Other', 'Lineage_Omicron' | 0.663017736 |
| Age', 'Sex', 'Healthcare worker', 'CCI', 'Diabetes', 'Hypertension', 'Obesity', 'Precipitation', 'Relative humidity', 'Wind speed', 'NO2', 'O3', 'PM10', 'PM2.5', 'Ethnic_White', 'Ethnic_Other', 'Lineage_Omicron' | 0.664575123 |
| Age', 'Sex', 'Healthcare worker', 'CCI', 'Diabetes', 'Hypertension', 'Obesity', 'Precipitation', 'Relative humidity', 'Wind speed', 'NO2', 'O3', 'PM10', 'PM2.5', 'Ethnic_White', 'Lineage_Omicron' | 0.661987343 |
| Age', 'Sex', 'Healthcare worker', 'CCI', 'Diabetes', 'Hypertension', 'Obesity', 'Precipitation', 'Relative humidity', 'Wind speed', 'NO2', 'O3', 'PM10', 'PM2.5', 'Ethnic_White' | 0.659936671 |

| ACO-COVID model |  |
| --- | --- |
| **Variable** | **AUC** |
| Age', 'Sex', 'Healthcare worker', 'CCI', 'Hypertension', 'Obesity', 'Temperature', 'Precipitation', 'Relative humidity', 'Wind speed', 'O3', 'Ethnic_Asian', 'Ethnic_Black', 'Ethnic_LatinAmerican', 'Ethnic_White', 'Ethnic_Other', 'Lineage_Alpha', 'Lineage_Beta', 'Lineage_Delta', 'Lineage_Gamma', 'Lineage_Omicron', 'Lineage_Other' | 0.819347768 |
| Age', 'Sex', 'CCI', 'Hypertension', 'Obesity', 'Temperature', 'Precipitation', 'Relative humidity', 'Wind speed', 'O3', 'Ethnic_White', 'Lineage_Beta', 'Lineage_Delta', 'Lineage_Omicron', 'Lineage_Other' | 0.814795856 |
| Age', 'Sex', 'CCI', 'Hypertension', 'Obesity', 'Temperature', 'Precipitation', 'Relative humidity', 'Wind speed', 'O3', 'Ethnic_White', 'Lineage_Delta', 'Lineage_Omicron', 'Lineage_Other' | 0.814834416 |
| Age', 'Sex', 'CCI', 'Hypertension', 'Obesity', 'Temperature', 'Precipitation', 'Relative humidity', 'Wind speed', 'O3', 'Ethnic_White', 'Lineage_Delta', 'Lineage_Other' | 0.812549085 |
| Age', 'Sex', 'CCI', 'Hypertension', 'Obesity', 'Temperature', 'Precipitation', 'Relative humidity', 'Wind speed', 'O3', 'Ethnic_White', 'Lineage_Other' | 0.814312257 |
| Age', 'Sex', 'CCI', 'Hypertension', 'Obesity', 'Temperature', 'Precipitation', 'Relative humidity', 'Wind speed', 'O3', 'Ethnic_White' | 0.811394745 |
| Age', 'CCI', 'Hypertension', 'Obesity', 'Temperature', 'Precipitation', 'Relative humidity', 'Wind speed', 'O3', 'Ethnic_White' | 0.812046383 |

Supplementary table 2. Contribution of factors to predicting admission severity among asthma-, CPD-, and ACO-COVID patients.

| **Category** | **Variable** | **Importance score** | | |
| --- | --- | --- | --- | --- |
|  |  | **Asthma-COVID** | **CPD-COVID** | **ACO-COVID** |
| **Individual factor** | Age | 0.305168878 | 0.268003803 | 0.291156972 |
|  | Sex | 0.110308406 | 0.08820813 | 0.071328017 |
|  | Ethnicity | 0.048217674 | 0.033008657 | 0.466885954 |
|  | COVID-19 vaccine | 0.053086182 | 0 | 0 |
|  | Healthcare worker | 0 | 0.024804441 | 0.010246374 |
|  | CCI index | 0.07145623 | 0.043851333 | 0.169928723 |
|  | Obesity | 0.188211969 | 0.092409566 | 0.484035049 |
|  | Hypertension | 0.042390402 | 0.042167636 | 0.070912651 |
|  | Diabetes | 0.024391136 | 0.030617565 | 0 |
| **Environmental factor** | Humidity | 0.073448352 | 0.126884471 | 0.200748394 |
|  | Temperature | 0.073430843 | 0 | 0.112454927 |
|  | Precipitation | 0.042147452 | 0.052512819 | 0.138098988 |
|  | Wind speed | 0 | 0.045509339 | 0.162768774 |
|  | PM_2.5_ | 0.047660509 | 0.021424649 | 0 |
|  | PM_10_ | 0.05776341 | 0.022172045 | 0 |
|  | NO_2_ | 0.03714922 | 0.031969024 | 0 |
|  | SO_2_ | 0.04115303 | 0 | 0 |
|  | O_3_ | 0.034344139 | 0.065779647 | 0.152832333 |
| **Viral variant** | Variant | 0.211766568 | 0.104440277 | 0.14882838 |

Supplementary table 3. Contribution of factors to predicting 28-day mortality among asthma-, CPD-, and ACO-COVID patients.

| **Category** | **Variable** | **Importance score** | | |
| --- | --- | --- | --- | --- |
|  |  | **Asthma-COVID** | **CPD-COVID** | **ACO-COVID** |
| **Individual factor** | Age | 0.753758526 | 0.347299221 | 0.445035148 |
|  | Sex | 0.044699613 | 0.132618853 | 0.100665033 |
|  | Ethnicity | 0.040780261 | 0 | 0 |
|  | COVID-19 vaccine | 0.055031825 | 0.068831475 | 0.033256063 |
|  | Healthcare worker | 0.048762305 | 0.032523581 | 0 |
|  | CCI index | 0.145875338 | 0.141589055 | 0.099792051 |
|  | Obesity | 0.101333058 | 0.024252636 | 0.025667054 |
|  | Hypertension | 0.027534698 | 0 | 0 |
|  | Diabetes | 0.017255228 | 0 | 0.052778862 |
| **Environmental factor** | Humidity | 0.056228491 | 0.022811421 | 0.00803299 |
|  | Temperature | 0 | 0.069804621 | 0 |
|  | Precipitation | 0 | 0.031726237 | 0.031726237 |
|  | Wind speed | 0.029789479 | 0.023095587 | 0 |
|  | PM2.5 | 0 | 0.012541915 | 0.057104954 |
|  | PM10 | 0.032017429 | 0.004762597 | 0.00688061 |
|  | NO2 | 0.041140554 | 0.037709346 | 0.040934766 |
|  | SO2 | 0.018317289 | 0.015589898 | 0.011053438 |
|  | O3 | 0 | 0.021081399 | 0.021896322 |
| **Viral variant** | Variant | 0.162090688 | 0.207436385 | 0.177740938 |

Supplementary table 4. The number of clusters based on silhouette coefficients

| **Number of clusters** | **Silhouette score** | | | | | |
| --- | --- | --- | --- | --- | --- | --- |
|  | Asthma-COVID | | CPD-COVID | | ACO-COVID | |
|  | Severe | Mild | Severe | Mild | Severe | Mild |
| 2 | 0.33 | 0.32 | 0.33 | 0.14 | 0.2 | 0.28 |
| 3 | 0.32 | 0.18 | 0.28 | 0.15 | 0.2 | 0.27 |
| 4 | 0.27 | 0.2 | 0.21 | 0.12 | 0.17 | 0.25 |
| 5 | 0.28 | 0.23 | 0.24 | 0.15 | 0.14 | 0.26 |

Supplementary table 5. Sensitivity analysis of the association between humidity and severity in asthma-COVID patients

| **Scenario** | **Coefficient** | **Standard deviation** | **OR** | **P value** |
| --- | --- | --- | --- | --- |
| Humidity>=60 | -0.07495069 | 0.01083231 | 0.9277892 | <0.001 |
| Humidity>=50 | -0.07986389 | 0.01000972 | 0.923242 | <0.001 |
| Humidity>=40 | -0.07818465 | 0.009526913 | 0.9247936 | <0.001 |
| All levels of humidity | -0.083172784 | 0.009618767 | 0.920192139 | <0.001 |

Supplementary table 6. Sensitivity analysis: exclusion of Q1 2020 data (asthma-COVID patients)

| **Variable** | **OR (primary analysis)** | **OR (sensitive analysis)** | **P value (primary analysis)** | **P value (sensitive analysis)** |
| --- | --- | --- | --- | --- |
| Age |  |  |  |  |
| 0~9 | 0.25 | 0.25 | <0.001 | <0.001 |
| 10~19 | 0.24 | 0.24 | <0.001 | <0.001 |
| 20~29 | 0.40 | 0.41 | <0.001 | <0.001 |
| 30~39 | 0.64 | 0.65 | <0.001 | <0.001 |
| 40~49 | 0.87 | 0.88 | <0.001 | <0.001 |
| 50~59 | 1.00 | 1.00 |  |  |
| 60~69 | 1.07 | 1.07 | 0.084 | 0.064 |
| 70~79 | 0.85 | 0.85 | <0.001 | <0.001 |
| 80~89 | 0.52 | 0.53 | <0.001 | <0.001 |
| >=90 | 0.44 | 0.46 | <0.001 | <0.001 |
| Sex |  |  |  |  |
| Male | 1.28 | 1.30 | <0.001 | <0.001 |
| Ethnicity |  |  |  |  |
| White | 1.00 | 1.00 |  |  |
| Black | 1.38 | 1.34 | <0.001 | <0.001 |
| Asian | 1.22 | 1.21 | <0.001 | <0.001 |
| Latin American | 1.38 | 1.27 | 0.241 | 0.398 |
| Other | 0.91 | 0.90 | 0.002 | 0.003 |
| COVID-19 vaccine |  |  |  |  |
| Yes | 0.76 | 0.76 | <0.001 | <0.001 |
| Healthcare worker |  |  |  |  |
| Yes | 1.00 | 1.03 | 0.949 | 0.672 |
| CCI index |  |  |  |  |
|  | 0.92 | 0.91 | <0.001 | <0.001 |
| Diabetes |  |  |  |  |
| Yes | 1.12 | 1.15 | <0.001 | <0.001 |
| Hypertension |  |  |  |  |
| Yes | 1.08 | 1.07 | 0.004 | 0.018 |
| Obesity |  |  |  |  |
| Yes | 1.85 | 1.87 | <0.001 | <0.001 |
| Smoking |  |  |  |  |
| Yes | 0.94 | 0.94 | 0.087 | 0.103 |
| Lineage |  |  |  |  |
| Other | 1.00 | 1.00 |  |  |
| Alpha | 1.26 | 1.31 | <0.001 | <0.001 |
| Delta | 0.99 | 1.02 | 0.750 | 0.656 |
| Omicron | 0.38 | 0.39 | <0.001 | <0.001 |
| Temperature |  |  |  |  |
| Per 5℃ | 0.92 | 0.99 | <0.001 | 0.001 |
| Precipitation |  |  |  |  |
| Per 10mm | 0.98 | 0.98 | <0.001 | <0.001 |
| Humidity |  |  |  |  |
| Per 5% | 0.92 | 0.98 | <0.001 | <0.001 |
| Wind speed |  |  |  |  |
| Per 1m/s | 0.98 | 0.90 | 0.372 | <0.001 |
| SO_2_ |  |  |  |  |
| Per 10ug/m^3^ | 1.12 | 1.01 | <0.001 | <0.001 |
| NO_2_ |  |  |  |  |
| Per 10ug/m^3^ | 1.15 | 1.02 | <0.001 | <0.001 |
| O_3_ |  |  |  |  |
| Per 10ug/m^3^ | 1.03 | 1.00 | 0.037 | 0.935 |
| PM_2.5_ |  |  |  |  |
| Per 10ug/m^3^ | 1.06 | 1.01 | <0.001 | <0.001 |
| PM_10_ |  |  |  |  |
| Per 10ug/m^3^ | 1.04 | 1.00 | <0.001 | <0.001 |

Supplementary table 7. Sensitivity analysis: exclusion of Q1 2020 data (CPD-COVID patients)

| **Variable** | **OR (primary analysis)** | **OR (sensitive analysis)** | **P value (primary analysis)** | **P value (sensitive analysis)** |
| --- | --- | --- | --- | --- |
| Age |  |  |  |  |
| 0~9 | 0.84 | 0.94 | 0.327 | 0.725 |
| 10~19 | 0.65 | 0.67 | 0.102 | 0.225 |
| 20~29 | 0.53 | 0.51 | 0.001 | 0.001 |
| 30~39 | 0.59 | 0.57 | <0.001 | <0.001 |
| 40~49 | 0.79 | 0.79 | 0.004 | 0.006 |
| 50~59 | 1 | 1.00 |  |  |
| 60~69 | 1.2 | 1.23 | <0.001 | <0.001 |
| 70~79 | 1.02 | 1.05 | 0.670 | 0.323 |
| 80~89 | 0.69 | 0.71 | <0.001 | <0.001 |
| >=90 | 0.56 | 0.56 | <0.001 | <0.001 |
| Sex |  |  |  |  |
| Male | 1.24 | 1.22 | <0.001 | <0.001 |
| Ethnicity |  |  |  |  |
| White | 1 | 1.00 |  |  |
| Black | 2.06 | 1.87 | <0.001 | <0.001 |
| Asian | 1.44 | 1.47 | <0.001 | <0.001 |
| Latin American | 1.16 | 0.96 | 0.638 | 0.926 |
| Other | 0.93 | 0.92 | 0.038 | 0.020 |
| COVID-19 vaccine |  |  |  |  |
| Yes | 1.04 | 1.02 | 0.492 | 0.663 |
| Healthcare worker |  |  |  |  |
| Yes | 1.51 | 1.58 | 0.001 | 0.001 |
| CCI index |  |  |  |  |
|  | 0.95 | 0.96 | <0.001 | <0.001 |
| Diabetes |  |  |  |  |
| Yes | 0.89 | 0.88 | <0.001 | <0.001 |
| Hypertension |  |  |  |  |
| Yes | 1.06 | 1.05 | 0.040 | 0.099 |
| Obesity |  |  |  |  |
| Yes | 1.76 | 1.82 | <0.001 | <0.001 |
| Smoking |  |  |  |  |
| Yes | 1.05 | 1.04 | 0.168 | 0.280 |
| Lineage |  |  |  |  |
| Other | 1 | 1.00 |  |  |
| Alpha | 0.89 | 0.92 | 0.003 | 0.031 |
| Delta | 0.85 | 0.88 | <0.001 | 0.001 |
| Omicron | 0.47 | 0.49 | <0.001 | <0.001 |
| Temperature |  |  |  |  |
| Per 5℃ | 1.01 | 1.01 | 0.790 | 0.032 |
| Precipitation |  |  |  |  |
| Per 10mm | 0.98 | 1.00 | <0.001 | <0.001 |
| Humidity |  |  |  |  |
| Per 5% | 0.92 | 0.98 | <0.001 | <0.001 |
| Wind speed |  |  |  |  |
| Per 1m/s | 0.93 | 0.81 | <0.001 | <0.001 |
| SO_2_ |  |  |  |  |
| Per 10ug/m^3^ | 1.05 | 1.01 | 0.132 | 0.070 |
| NO_2_ |  |  |  |  |
| Per 10ug/m^3^ | 0.93 | 1.00 | 0.033 | 0.334 |
| O_3_ |  |  |  |  |
| Per 10ug/m^3^ | 1.12 | 1.01 | <0.001 | <0.001 |
| PM_2.5_ |  |  |  |  |
| Per 10ug/m^3^ | 1.04 | 1.00 | 0.004 | 0.003 |
| PM_10_ |  |  |  |  |
| Per 10ug/m^3^ | 1.03 | 1.00 | 0.005 | 0.004 |

Supplementary table 8. Sensitivity analysis: exclusion of Q1 2020 data (ACO-COVID patients)

| **Variable** | **OR (primary analysis)** | **OR (sensitive analysis)** | **P value (primary analysis)** | **P value (sensitive analysis)** |
| --- | --- | --- | --- | --- |
| Age |  |  |  |  |
| 0~9 | 0.06 | 0.07 | <0.001 | <0.001 |
| 10~19 | 0.25 | 0.25 | 0.058 | 0.059 |
| 20~29 | 0.37 | 0.40 | 0.004 | 0.009 |
| 30~39 | 0.61 | 0.61 | 0.009 | 0.012 |
| 40~49 | 0.73 | 0.73 | 0.018 | 0.021 |
| 50~59 | 1 | 1.00 |  |  |
| 60~69 | 1.24 | 1.21 | 0.008 | 0.028 |
| 70~79 | 1.01 | 1.02 | 0.870 | 0.809 |
| 80~89 | 0.72 | 0.73 | <0.001 | 0.001 |
| >=90 | 0.59 | 0.60 | <0.001 | 0.001 |
| Sex |  |  |  |  |
| Male | 1.22 | 1.20 | <0.001 | <0.001 |
| Ethnicity |  |  |  |  |
| White | 1 | 1.00 |  |  |
| Black | 1.86 | 1.99 | 0.002 | 0.001 |
| Asian | 1.15 | 1.17 | 0.286 | 0.274 |
| Latin American | 1.17 | 0.23 | 0.863 | 0.301 |
| Other | 0.81 | 0.78 | 0.004 | 0.002 |
| COVID-19 vaccine |  |  |  |  |
| Yes | 0.9 | 0.91 | 0.340 | 0.392 |
| Healthcare worker |  |  |  |  |
| Yes | 1.75 | 1.83 | 0.014 | 0.010 |
| CCI index |  |  |  |  |
|  | 0.9 | 0.90 | <0.001 | <0.001 |
| Diabetes |  |  |  |  |
| Yes | 1.02 | 1.03 | 0.767 | 0.648 |
| Hypertension |  |  |  |  |
| Yes | 1.11 | 1.10 | 0.048 | 0.101 |
| Obesity |  |  |  |  |
| Yes | 2.03 | 2.12 | <0.001 | <0.001 |
| Smoking |  |  |  |  |
| Yes | 0.97 | 0.98 | 0.664 | 0.792 |
| Lineage |  |  |  |  |
| Other | 1 | 1.00 |  |  |
| Alpha | 0.96 | 0.97 | 0.558 | 0.747 |
| Delta | 0.71 | 0.71 | <0.001 | <0.001 |
| Omicron | 0.62 | 0.63 | <0.001 | <0.001 |
| Temperature |  |  |  |  |
| Per 5℃ | 1.12 | 1.03 | 0.003 | <0.001 |
| Precipitation |  |  |  |  |
| Per 10mm | 0.98 | 1.00 | 0.022 | 0.103 |
| Humidity |  |  |  |  |
| Per 5% | 0.92 | 0.98 | <0.001 | <0.001 |
| Wind speed |  |  |  |  |
| Per 1m/s | 0.88 | 0.79 | 0.004 | <0.001 |
| SO_2_ |  |  |  |  |
| Per 10ug/m^3^ | 0.96 | 1.00 | 0.462 | 0.373 |
| NO_2_ |  |  |  |  |
| Per 10ug/m^3^ | 0.91 | 0.99 | 0.157 | 0.190 |
| O_3_ |  |  |  |  |
| Per 10ug/m^3^ | 1.12 | 1.01 | <0.001 | 0.004 |
| PM_2.5_ |  |  |  |  |
| Per 10ug/m^3^ | 0.98 | 1.00 | 0.468 | 0.382 |
| PM_10_ |  |  |  |  |
| Per 10ug/m^3^ | 0.99 | 1.00 | 0.433 | 0.340 |
